# Long-term relationships between summer clouds and aerosols over mid-high latitudes of the Northern Hemisphere

**DOI:** 10.1038/s41598-024-59817-7

**Published:** 2024-04-20

**Authors:** Akihisa Watari, Yoshinori Iizuka, Koji Fujita, Hirohiko Masunaga, Kazuaki Kawamoto

**Affiliations:** 1 Graduate School of Environmental Science, Hokkaido University, Sapporo, 060-0810 Japan; 2 Institute of Low Temperature Science, Hokkaido University, Sapporo, 060-0819 Japan; 3 Graduate School of Environmental Studies, Nagoya University, Nagoya, 464-8601 Japan; 4 Institute for Space-Earth Environmental Research, Nagoya University, Nagoya, 464-8601 Japan; 5 Graduate School of Fisheries and Environmental Sciences, Nagasaki University, Nagasaki, 852-8521 Japan; 6 Present Address: Nippon Koei Energy Solutions Co., Ltd, Tokyo, 102-8539 Japan

**Keywords:** Climate sciences, Environmental sciences, Chemistry

## Abstract

While the short-term relationship between clouds and aerosols is well known, no adequate data is available to verify the longer-term, annual to decadal, relationship. It is important to quantify the aerosol–cloud interaction (ACI) for mitigating uncertainty in climate prediction. Here the long-term ACI over the mid-to-high latitudes of the Northern Hemisphere was analyzed by using seasonally-resolved ion fluxes reconstructed from a southeastern Greenland ice core (SE-Dome ice core) as aerosol proxies, and satellite-based summer cloud amount between 1982 and 2014. As a result, SO_4_^2−^ flux in the ice core shows significant positive correlation with total cloud amounts (${CC}_{T}$) and cloud droplet concentration (${N}_{d}$) in the summer over the southeastern Greenland Sea, implying that the sulfate aerosols may contribute to the variability of ${CC}_{T}$ via microphysical cloud processes. Significant positive correlations are persistent even under the constrained conditions when cloud formation factors such as relative humidity, air temperature at cloud height, and summer North Atlantic Oscillation are limited within ± 1σ variability. Hence sulfate aerosols should control the interannual variability of summer ${CC}_{T}$ In terms of decadal changes, ${CC}_{T}$ was approximately 3–5% higher in the 1960s–1970s than in the 1990s–2000s, which can be explained by changes in the, ${{{\text{SO}}}_{4}}^{2-}$ flux preserved in the SE-Dome ice core.

## Introduction

Aerosol cloud-mediated radiative effects are thought to exert a significant cooling effect on the Earth’s climate. However, quantifying the aerosol–cloud interaction (ACI) has been a major challenge despite scientific efforts over the past few decades^1,2^. Estimation of aerosol-induced cloud amounts, called the “Albrecht effect”, is important in climate prediction^3^. In the past, ACI has been determined based on hourly to daily field measurements. For example, a study showed that the arctic environment had a high sensitivity to light-absorbing aerosols, such as black carbon, by heating the surrounding atmosphere and leading to the evaporation of low-level clouds, called the semi-direct effect^4^. It was also reported that lightning density was enhanced by up to a factor of two over shipping lanes compared to adjacent areas due to the additional aerosols from ships^5^. However, the annual to decadal ACI is poorly understood despite its crucial role in the climate system. Bellouin et al.^6^, reviewed aerosols interact with radiation and clouds that substantial progress made over the past 40 years in observing, understanding, and modeling these processes helped quantify the imbalance in the Earth’s radiation budget caused by anthropogenic aerosols, called aerosol radiative forcing, but uncertainties remain large. Also, they pointed out a necessary of local process studies that the lack of resolution of small scales by large-scale models means that their integration of local processes into a globally averaged number is imperfect. Only model studies are used for historical experiments from industrial revolution to present, primarily because of the lack of aerosol and cloud observations. Satellites are the best source of historical cloud observations since 1980s, and past aerosols are preserved in polar ice cores. The direct comparison with satellite-based relationships of clouds and aerosols has been difficult due to the lack of reliable long-term datasets, especially focused on aerosol species likely to be cloud nuclei. In reanalysis of cloud data used in previous studies, it has been reported that there are large uncertainties compared to satellite and field data^7,8^, thus the use of reanalysis data is not optimal for a detailed comparison between clouds and aerosols.

The Arctic near-surface temperature continues to rise at double the rate of global average values, which have been reported by field observation and models^9,10^; a phenomenon called Arctic amplification. Ice cores drilled from the Greenland ice sheet can provide fluctuation records of past aerosols in the Arctic^11,12^. In particular, an ice core from the southeastern dome of Greenland has well-preserved aerosols on a seasonal-scale with less post-depositional loss under high accumulation rates (~ 1.0 m w.e. a^−1^), and can track the variability of seasonal aerosol fluxes for the long-term (1960–2014) atmospheric environment^13,14^.

Here, long-term ACI was investigated using the new satellite-based cloud datasets (1982–2016), called Cloud_cci (CCI) data, and the SE-Dome ice core data over the mid-to-high latitudes of the Northern Hemisphere. The historical variability of aerosol-driven cloud amounts in summer (Jun, July, and August; JJA) are evaluated over the 33 year spanning 1982–2014. This period includes a time of high-pollution including significant emissions of anthropogenic aerosols after the 1980s, some aerosol proxies decreased by the regulation of anthropogenic activity^15^. The CCI is based on advanced very high-resolution radiometer (AVHRR) post meridiem version 3^16,17^, which contained various cloud properties for the period from 1982 to 2016. The datasets are consistently retrieved from AVHRR, which is one of the oldest cloud satellites^18^, combined with some passive cloud datasets to achieve increased temporal resolution with high spatial resolution covering the whole globe.

### SO_4_^2−^ flux and cloud properties

**Figure 1:**
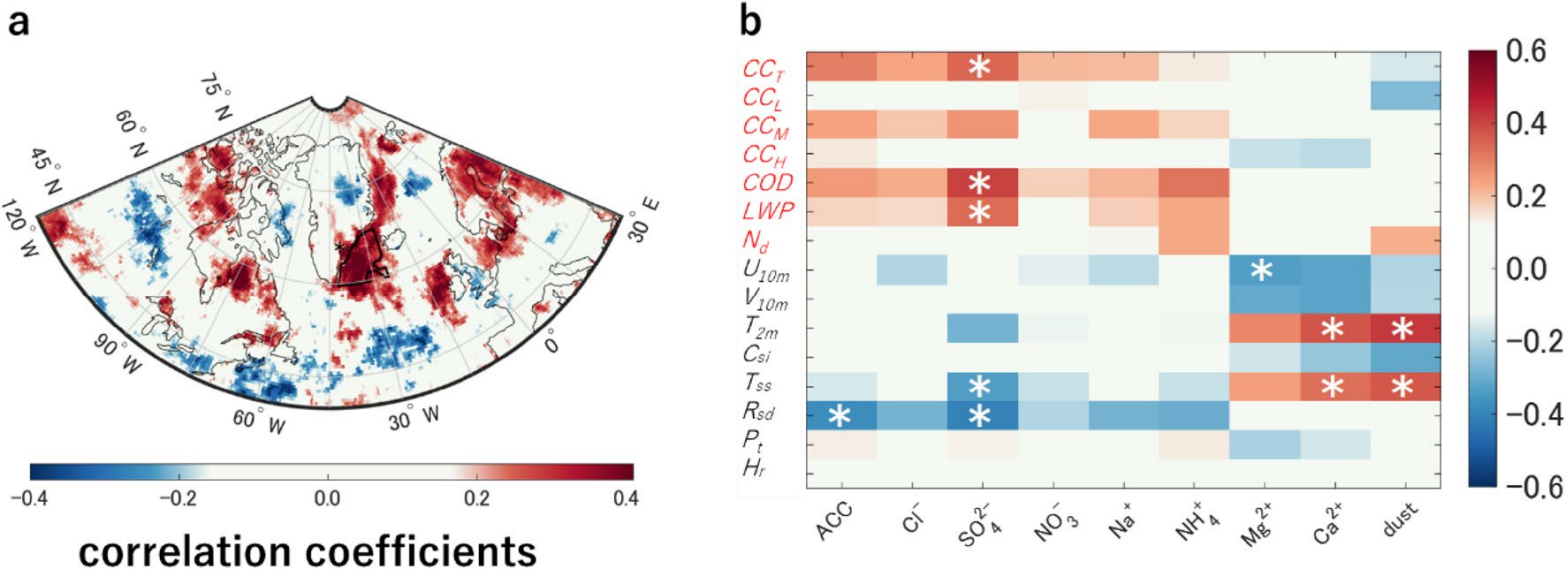
Relationships of aerosol proxies in the SE-Dome ice core and cloud properties. (**a**) Geographical distribution of correlation coefficients between SO_4_^2−^ flux and ${CC}_{T}$ in summer with a resolution of 0.5º from 1982 to 2014. The black line shows the target domain for the correlation analysis. An asterisk denotes the SE-Dome site where the ice core was drilled^13^. (**b**) The area-averaged correlation coefficients between aerosol proxies preserved in the SE-Dome ice core and cloud and atmospheric properties in the target domain. The vertical axis shows the cloud properties (red text); total cloud amounts (${CC}_{T}$), low level (${CC}_{L}$), middle level (${CC}_{M}$), and high level (${CC}_{H}$), cloud optical depth ($COD$), liquid water path ($LWP$), and cloud droplet concentration (${N}_{d}$), and the atmospheric properties (black text); wind components at 10 m height (${U}_{10m}$ and ${V}_{10m}$), 2 m height air temperature (${T}_{2m}$), sea ice concentration (${C}_{si}$), sea surface temperature (${T}_{ss}$), downward solar radiation at the surface (${R}_{sd}$), total precipitation (${P}_{t}$), and relative humidity (${H}_{r}$), respectively. The horizontal axis shows the aerosol proxies reconstructed in the SE-Dome ice core. White asterisks in panel b denote *p* < 0.05.

First, the geographical distribution of correlation coefficients between the aerosol proxies (SO_4_^2−^, NO_3_^−^, Cl^−^, Na^+^, NH_4_^+^, Mg^2+^, Ca^2+^, and dust) and cloud properties (total cloud amounts (${CC}_{T}$), low level (${CC}_{L}$), middle level (${CC}_{M}$), and high level (${CC}_{H}$), cloud optical depth ($COD$), liquid water path ($LWP$), and cloud droplet concentration (${N}_{d}$) ) are analyzed for all seasons (spring (March, April, and May; MAM), summer (JJA), autumn (September, October, and November; SON), and winter (December, January, and February; DJF)) from 1982 to 2014 (Figs. S1–S25). Among them, we focused on ${{{\text{SO}}}_{4}}^{2-}$ flux in the summer because (1) it has the most relevant correlations to cloud properties, (2) summer should be the season with the most solar radiation and also clouds, and should influence the polar climate, and (3) sulfate aerosols are reported to be the highest cloud albedo effects among the aerosols^19^. Figure 1a shows that the summer ${{{\text{SO}}}_{4}}^{2-}$ flux at the SE-Dome site significantly correlated with ${CC}_{T}$ over the southeast Greenland ocean where the probability of air mass to the SE-Dome site were more than 50% (area surrounded by black line in Fig. S26 based on 14 days backtrajectory^14^, also see “Methods”), implying that summer ${{{\text{SO}}}_{4}}^{2-}$ flux could be a proxy of ${CC}_{T}$ over the ocean.

In order to make clear the marine cloud over a target domain (blacklines in Fig. 1a, 25º–45ºW and 60º–68ºN, modified from Fig. S26), the averaged correlation coefficients of the aerosol proxies and cloud properties over the target domain in all seasons from 1982 to 2014 (Fig. S27) are investigated to use SO_4_^2−^ flux as a proxy for the past cloud amounts in summer. Although most aerosols proxies have no significant correlation with cloud properties, some significant correlations are found (asterisks in Fig. S27). For instance, a negative correlation between dust flux and ${CC}_{L}$ due to the suppression of dust nucleation when a certain amount of dust aerosol is present in the atmosphere is found. These correlations were reported in previous studies^20^. Among aerosols, summer ${{{\text{SO}}}_{4}}^{2-}$ flux is the most relevant to ${CC}_{T}$ in the target domain (Fig. 1b). The ${{{\text{SO}}}_{4}}^{2-}$ flux also significantly correlates with the downward solar radiation at the surface (${R}_{sd}$, *r* = –0.49, *p* < 0.01), cloud optical depth ($COD$, *r* = 0.42, *p* < 0.05), and liquid water path ($LWP$, *r* = 0.34, *p* < 0.05) (Fig. 1b). A previous study suggested that polluted marine clouds could increase in $LWP$ due to growth in optically thick clouds by analyzing stratocumulus along shipping lanes^21^. Other studies have shown that sulfate aerosol particles could act as cloud condensation nuclei (*CCN*) in the cloud formation processes^22,23^. ${{{\text{SO}}}_{4}}^{2-}$ flux in the SE-Dome ice core is highly correlated with *cloud amounts* which varies with the *COD*, suggesting that summer SO_4_^2−^ flux contributes to marine cloud formation over the domain.

The correlation plots among the aerosol proxies show that ${{{\text{SO}}}_{4}}^{2-}$ and ${{{\text{NO}}}_{3}}^{-}$ fluxes highly correlate in summer (Fig. S28b, *r* = 0.76, *p* < 0.001), which is roughly consistent with emission sources and deposition processes, while the correlation coefficient between the NO_3_^−^ flux but ${CC}_{T}$ in the target domain was not significant (*r* = 0.22, *p* = 0.37)^24^. These results and discussion suggest that ${{{\text{SO}}}_{4}}^{2-}$ flux is cloud-related more than the NO_3_^−^ flux by efficiently serving as $CCN$^22^.

For reference, ${{{\text{SO}}}_{4}}^{2-}$ flux also shows significant correlation with the cloud amounts in the ERA5 reanalysis data for the same domain (*r* = 0.36, *p* < 0.05 for ${CC}_{L}$ and *r* = 0.38, *p* < 0.05 for ${CC}_{T}$, Fig. S29) and their distributions are qualitatively similar to the satellite-based results (Fig. 1a).

### ${{SO}_{4}}^{2-}$ flux and cloud droplet concentration

**Figure 2:**
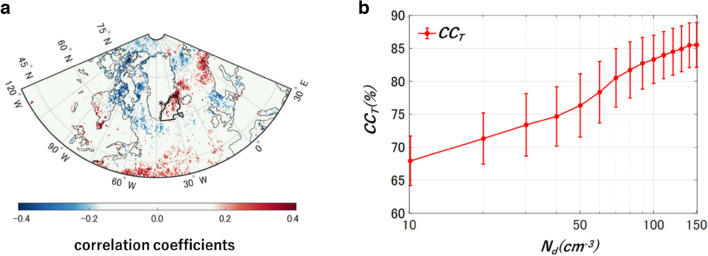
${N}_{d}$ have positive correlation with SO_4_^2−^ flux and ${CC}_{T}$ in the target domain. (**a**) Geographical distribution of correlation coefficients between SO_4_^2−^ flux and ${N}_{d}$ for low-level warm clouds with a resolution of 0.5º from 1982 to 2014. (**b**) Dependence of ${CC}_{T}$ on ${N}_{d}$ in the target domain (black polygon in panel a). Error bars represent 1 standard deviation of the monthly ${CC}_{T}$.

The satellite-based ${N}_{d}$ (also see “Methods”)^25^ is calculated to investigate the cloud formation influenced by sulfate aerosols. Figure 2a shows the spatial distribution of correlation coefficients between ${{{\text{SO}}}_{4}}^{2-}$ flux and ${N}_{d}$ for low-level warm clouds. Positive correlations were observed in a portion of the target domain, even though the area-averaged correlation coefficients were not significant. This positive correlation is consistent with that between the ${{{\text{SO}}}_{4}}^{2-}$ flux and ${CC}_{T}$ (Fig. 1a). The variability of sulfate aerosol leads to changing ${N}_{d}$ loads in the target domain. ${N}_{d}$ has been used as a proxy for $CCN$ concentrations and has a remarkable power-law relation to the corresponding aerosol optical depth ($AOD$)^25,26^. For instance, cloud amounts over the ocean increase with both $LWP$ and ${N}_{d}$ until reaching nearly total cloud amounts for a given cloud geometrical thickness^27^. The clouds precipitate substantially, whereas precipitation in clouds is mostly suppressed as a result of the smaller cloud droplet size when ${N}_{d}$ increases. Figure 2b shows the dependence of ${CC}_{T}$ on ${N}_{d}$ in the target domain. Therefore, significantly positive correlations between ${{{\text{SO}}}_{4}}^{2-}$ flux and ${N}_{d}$ suggest a physically persistent process of ACI, which is clarified through a series of ${{{\text{SO}}}_{4}}^{2-}$ aerosol, ${N}_{d}$, and ${CC}_{T}$ in the target domain.

### SO_4_^2−^ flux and cloud amount under the constrained condition

**Figure 3:**
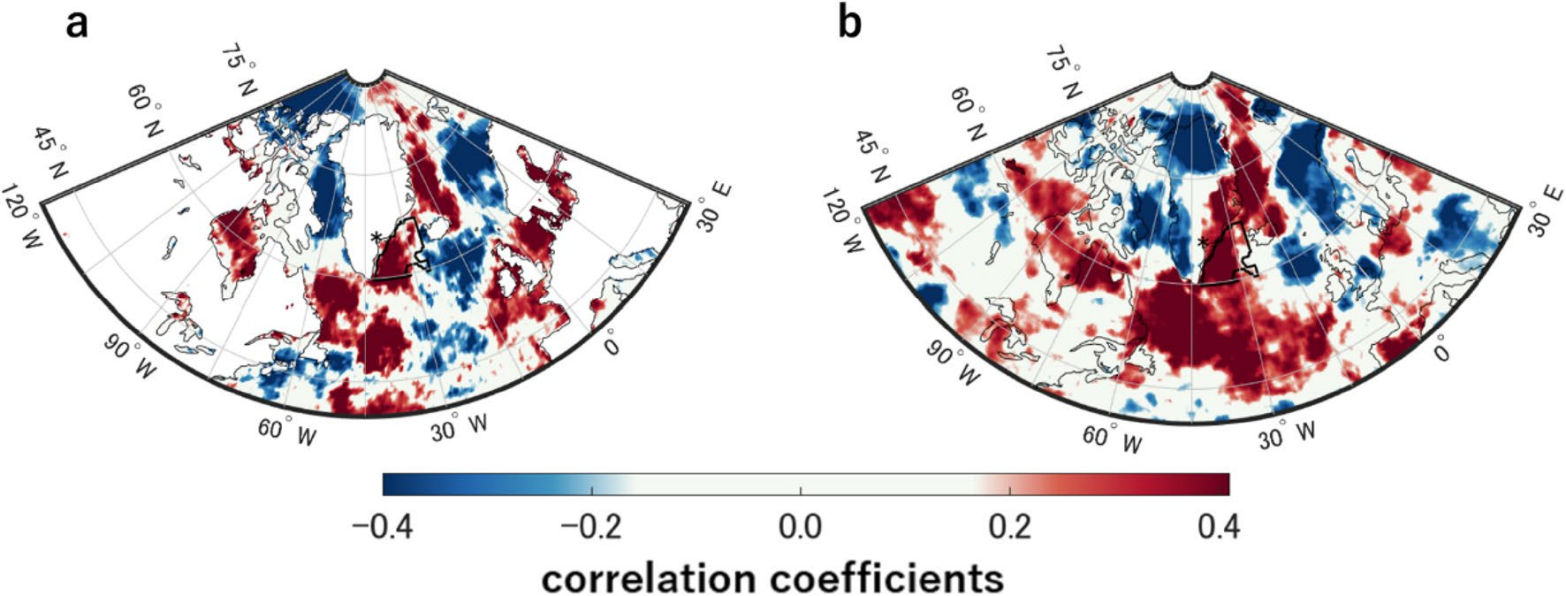
Relationships between ${CC}_{T}$ and SO_4_^2−^ flux under the constrained atmospheric conditions for cloud formation. Geographical distributions of the correlation coefficients between SO_4_^2−^ flux and ${CC}_{T}$ of (**a**) CCI and (**b**) ERA5 datasets under the constrained atmospheric conditions (${T}_{cloud\_height}$, ${H}_{r}$, and *sNAO* within <  ± 1σ) in the target domain.

Primary cloud formation depends on a variety of atmospheric variables such as temperature (${T}_{cloud\_height}$) and relative humidity (${H}_{r}$)^28,29^. Atmospheric circulation represented by climate indices (e.g., North Atlantic Oscillation (NAO), Arctic Oscillation (AO), Atlantic Multidecadal Oscillation (AMO)) is also important for cloud formation and distribution in mid-high latitudes of the Northern Hemisphere^30^. To avoid these effects from implicitly overriding the correlation between ${{{\text{SO}}}_{4}}^{2-}$ flux and ${CC}_{T}$, the meteorological conditions which could affect atmospheric circulation (${H}_{r}$, ${T}_{cloud\_height}$, and NAO index in summer; *sNAO*, Fig. S30 and see “Methods”) are constrained by limiting all variables within ± 1 standard deviation (14 of 33 years remained). Figure 3 shows the geographical distribution of the significant correlation between the ${{{\text{SO}}}_{4}}^{2-}$ flux and ${CC}_{T}$ (*r* > 0.49, *p* < 0.05) under the constrained conditions. Similar distributions of correlations with CCI and ERA5 data, and the persistent positive correlations in the target domains (Figs. 1a, 2a and 3). These results suggest cloud amount in the target domain varied positively with the sulfate aerosol flux to the SE-Dome site regardless of atmospheric conditions for main cloud formation (${H}_{r}$, ${T}_{cloud\_height}$, and *sNAO*).

### Past cloud amounts reconstruction

In this research, ${{{\text{SO}}}_{4}}^{2-}$ flux showed a positive correlation with satellite-based *N*_*d*_ in the target domain, which also supports a physically persistent process of ACI (Fig. 2). The sulfate aerosol preserved in the SE-Dome ice core in 1984 (17.32 $\mathrm{mg }{{\text{m}}}^{-2} {{\text{season}}}^{-1}$) and 1987 (10.82 ${\text{mg}}{\mathrm{ m}}^{-2} {{\text{season}}}^{-1}$) was more than two standard deviations higher between 1982 and 2014 (Fig. S31, 3.74 ± 3.23 mg m^−2^ season^–1^). Figure S32 shows the distributions of ${CC}_{T}$ and ${N}_{d}$ anomalies in 1984 and 1987 against the means (1982–2014). The mean ${CC}_{T}$ and ${N}_{d}$ in the target domain from 1982 to 2014 were 82.7 ± 7.8% and 94.0 ± 14.7 cm^−3^, respectively, while the ${CC}_{T}$ and ${N}_{d}$ anomalies were observed to be up to 8.9% and 59.1 cm^−3^. The observed changes in ${CC}_{T}$ and ${N}_{d}$ can be attributed to the increased ${{{\text{SO}}}_{4}}^{2-}$ flux at the SE-Dome site. The atmospheric transport processes from the emission sources to the SE-Dome site should have accounted for the higher ${{{\text{SO}}}_{4}}^{2-}$ fluxes. During the air mass transportation, the higher ${{{\text{SO}}}_{4}}^{2-}$ could have increased the cloud amount.

**Figure 4:**
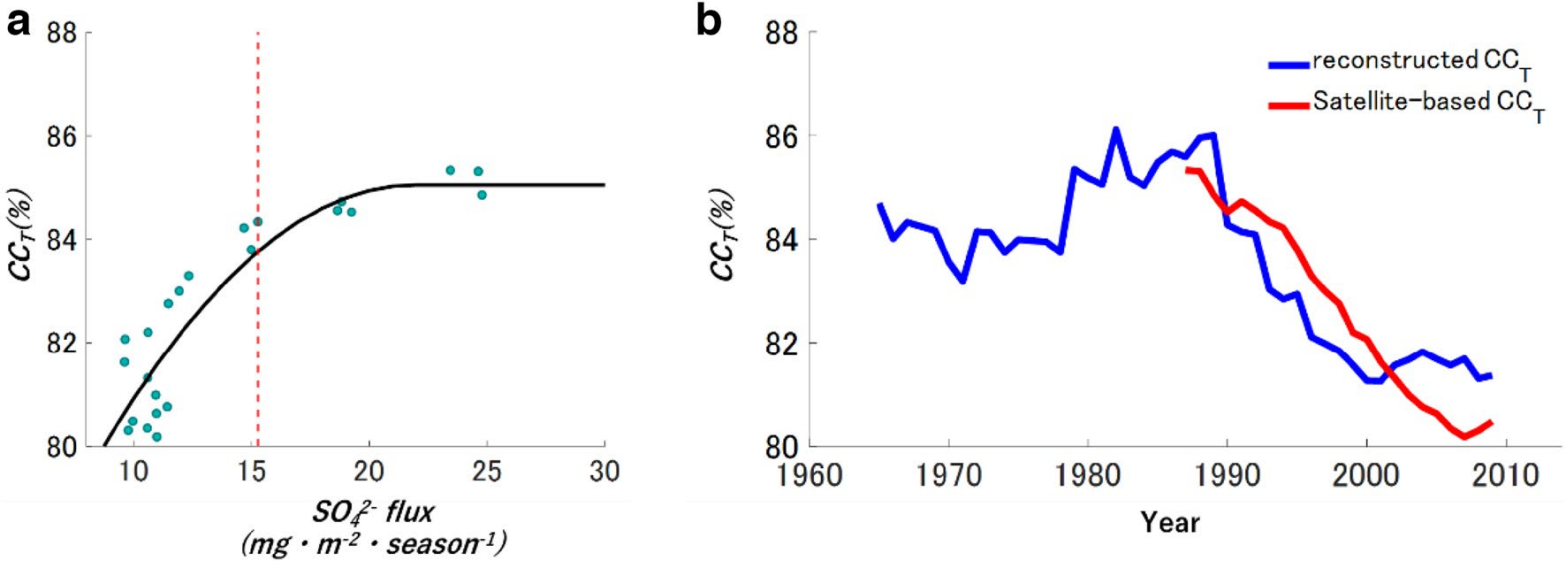
Reconstruction of *CC*_*T*_ from the SO_4_^2−^ flux in ice-core. (**a**) The scatter plot of the ${CC}_{T}$ and SO_4_^2−^ flux from 1982 to 2014 with a quadratic approximation (black line). The dashed red line indicates the value of 15.28 mg m^−2^ season^−1^ in SO_4_^2−^ flux. (**b**) An 11 year running mean of reconstructed ${CC}_{T}$ from the SE-Dome ice core (blue line) and satellite-based ${CC}_{T}$ in the CCI dataset averaged for the target domain (red line).

The relationship between 11 year-averaged ${{{\text{SO}}}_{4}}^{2-}$ flux (${F}_{{\text{SO}}4}$) and satellite-based ${CC}_{T}$ (%) are obtained for the 33 year period from 1982 to 2014 (Fig. 4a) to reconstruct past cloud amounts:

1 $$ CC_{T} = - 0.025F_{{{\text{SO}}_{4} }}^{2} + 1.272F_{{{\text{SO}}_{4} }} + 71.100 \,\left[ {F_{{{\text{SO}}_{4} }} < 25.44} \right] $$

2 $$ CC_{T} = 87.280 \,\left[ {F_{{{\text{SO}}_{4} }} \ge 25.44} \right] $$

Figure 4b shows the trends of the satellite-based ${CC}_{T}$ reconstruction from 1960 to 2014. The anthropogenic ${{\text{SO}}}_{2}$ during the highly polluted period should have contributed to the increases of sulfate aerosol particles^31^.

The reconstructed ${CC}_{T}$ in the 1960s and 1970s was approximately 3–5% higher than after 1991, which is similar to the ${CC}_{T}$ trends observed in Italy though the authors assumed that the trends should correlate with the NAO driven atmospheric circulation pattern^30^. Decreasing trends during the 1990s and a rather stable twenty-first century are consistent with those of satellite-based ${CC}_{T}$^30,32^. Thus, the relationship between averaged ${{{\text{SO}}}_{4}}^{2-}$ flux and reconstructed ${CC}_{T}$ suggests that the cloud amount over the ocean may have increased during 1960–80 via the aerosol Albrecht effect^3^. Also, the absence of a clear correlation for small values of ${{{\text{SO}}}_{4}}^{2-}$ flux in the relationship between averaged ${{{\text{SO}}}_{4}}^{2-}$ flux and satellite-based ${CC}_{T}$ for 55 years suggests a threshold of 15.28 mg m^−2^ season^−1^ due to the highest correlation to increases cloud amount effectively. This implies that an increase in aerosols does not directly cause an increase in amount of cloud, but that a certain amount of aerosols is required for a substantial increase in cloud amount. The concept of a threshold may be important in reconstructing cloud amounts over a wide area, which is required for further comparison between ice core data and satellite (or reanalysis) measurements, and would lead to improvement of physical mechanisms in aerosol-climate models.

The second ice core in the SE-Dome region is drilled in 2021, and the ice core cover pre-industrial revolution to present^33,34^, so that the new ice core will extend the interpretation obtained in this study to pre-industrial times. Bellouin et al.^6^, pointed out in the review that the degree to which human activities affect natural aerosol levels, and the response of clouds, and especially ice clouds, to aerosol perturbations remain particularly uncertain. The present paper reconstructs ${CC}_{T}$ during 1960–70 in Greenland, when the ${{\text{SO}}}_{x}$ emission maximum from around countries. The relationship between ${{{\text{SO}}}_{4}}^{2-}$^-^ aerosols and ${N}_{d}$ (and ${CC}_{T}$) during the ${{\text{SO}}}_{{\text{x}}}$ emission maximum will help to reduce the uncertainty in projections of future aerosols interacting with radiation and clouds through an improvement of climate models.

## Conclusions

The historical variability of aerosol-driven cloud amounts in summer were evaluated over a 33 year period from 1982 to 2014. Although most aerosol proxies have no significant correlation with cloud properties, ${{{\text{SO}}}_{4}}^{2-}$ flux has a highly positive correlation with cloud amount in the target domain. Positive correlations between SO_4_^2−^ flux and *N*_*d*_ were observed in a part of the target domain. The analyses suggest a physically persistent process of ACI, which is clarified through a series of ${{{\text{SO}}}_{4}}^{2-}$ aerosol, *N*_*d*_ and ${CC}_{T}$ in the target domain. Then, the meteorological conditions which could affect the atmospheric circulation (*H*_*r*_, *T*_*_cloud_height*_, and *sNAO*) were constrained for detailed analysis of the relationships between ${{{\text{SO}}}_{4}}^{2-}$ flux and ${CC}_{T}$. Regardless of the atmospheric conditions for cloud formation, ${{{\text{SO}}}_{4}}^{2-}$ has significant correlation with both satellite-based and reanalysis ${CC}_{T}$. Thus, ${{{\text{SO}}}_{4}}^{2-}$ flux preserved in the SE-Dome ice core cloud be a proxy of past cloud amounts. From significant correlations between ${{{\text{SO}}}_{4}}^{2-}$ flux and the cloud amounts from 1960 to 2014, it is estimated that ${CC}_{T}$ in the 1960s and 1970s was approximately 3–5% higher than that after 1991, which is similar to trends observed in Italy^30^. These results supported the Twomey effects, which implied the aerosols have driven the cloud microphysics process, especially in cloud amounts^3^.

## Methods

### Backward trajectory analysis

The transport pathways of air masses in the SE-Dome were analyzed according to Iizuka et al.^14^, who used the hybrid single-particle Lagrangian integrated trajectory (HYSPLIT model), which is distributed by NOAA (National Oceanic and Atmospheric Administration)^35^. The probability was weighted by the daily precipitation rate when the air mass arrived at the SE-Dome, because aerosols preserved in the ice core were wet deposited^14^. Figure S26 shows the probability distribution of air mass arriving at the SE-Dome site over 14 days for 1500 m above ground level in JJA from 1982 to 2014. The black line shows the area where the integrated probability of air mass at the SE-Dome was more than 50%.

### The main factors of cloud formation in the target domain

To exclude the influence of factors other than aerosols, the correlation coefficients between ${CC}_{T}$ and atmospheric environment (i.e. atmospheric pressure (*P*_*s*_), *H*_*r*_, *T*_*cloud_height*_, *C*_*si*_, *R*_*sd*_, wind direction (*D*_*W*_), wind speed (*S*_*W*_), *sNAO*, *GBI*, and *AMO*) are investigated. Due to the relative correlation among each variable, ${CC}_{T}$ in the target region have a high correlation with all atmospheric environments. For further analysis, the atmospheric environments that affect *CC*_*T*_ from previous research^28,29^ are considered. As a result, ${H}_{r}$, ${T}_{cloud\_height}$ in the cloud height (300–900 hpa), and *sNAO* indices are most relevant to cloud amount (Fig. S30). Next, the standard deviation of ${H}_{r}$, ${T}_{cloud\_height}$ and *sNAO* index are calculated over 33 years in the target domain where the relationship between ${{{\text{SO}}}_{4}}^{2-}$ flux and ${CC}_{T}$ was significant. To exclude the influence of factors other than aerosols, the period during which the all variables were within ± 1σ (dots in Fig. S30) are constrained. If the correlation coefficient between ${{{\text{SO}}}_{4}}^{2-}$ flux and cloud amount is still significant even under the constrained conditions of cloud formation factors (14-period), the variability of cloud amounts can be explained by the variability of aerosol concentrations.

### SE-Dome ice core data

The ice core used in this research was obtained at SE-Dome (67.18°N, 36.37°W, 3160 m a.s.l.) in southeast Greenland in 2015^14^. Due to its high accumulation rate (1.01 m w.e. a^−1^), the time scale of the SE-Dome ice core was determined to be from 1960 to 2014 with a measurement error of ± 2 months using the oxygen-isotope matching method^13,14^. The high temporal resolution of the aerosol proxies was reconstructed with better quality than any other ice cores drilled inside the ice sheets^14^. The high accumulation rate also allows aerosols to be preserved without post-depositional alternation^14^. The fluxes of SO_4_^2−^, NO_3_^−^, Cl^−^, Na^+^, NH_4_^+^, Mg^2+^, and Ca^2+^ as proxies for past aerosols were obtained with ion chromatography, and the dust was observed by a Beckman Coulter Counter Multisizer 3^13,14^. The measurement errors were reported to be 10% for the ions, and 15% for the dust^36^. In this research, aerosol fluxes are obtained using the accumulation rate of the SE-Dome ice core multiplied by the ion concentration.

### Cloud and meteorological data

Cloud datasets were obtained from the latest version of the advanced very high resolution radiometer post meridiem (AVHRR-PM) cloud data record (CDR), generated within the cloud component of the European Space Agency’s (ESA) climate change initiative programme (CCI) (i.e. Cloud_cci project). In the ESA Cloud_cci project, long-term and coherent cloud property datasets have been provided by exploiting the synergic capabilities of different earth passive satellites. Cloud detection and cloud property retrieval are done using the community cloud retrieval for climate (CC4CL) algorithm^17,18^. The CCI datasets from 1982 to 2014 were used in this research, providing sufficient samples to obtain statistically significant results of long-term ACI. The advantage of the CC4CL algorithm is that the visible infrared imager (AVHRR) has a proven track record of long-term observations and is useful for climate research. On the other hand, the disadvantage is that it is a passive sensor, so information on the vertical structure of clouds is limited, and so estimation of overlapping clouds is subject to large errors.

The meteorological data were used from ERA5 provided by the European Centre for Medium-Range Weather Forecasts (ECMWF). The ERA5 datasets are well-suited to understand the interactions of meteorological factors over the target region, due to the long time period and high spatial resolution. Supplementary Table 1 summarizes cloud properties in CCI and ERA5 used in this research.

### Methodology for calculating cloud droplet concentration

For each ${0.5}^{^\circ }\times {0.5}^{^\circ }$ grid, the ${N}_{d}$ over the ocean was calculated using cloudy pixels with $COD$ greater than the 90th percentile of cloud optical thickness ($\tau$) in $10\times 10$ pixels^25^. ${N}_{d}$ was used as a proxy of $CCN$ concentrations which depends on the aerosol loads and the updraft velocity^37^.

$${N}_{d}={C}^\frac{1}{2} {\tau }^\frac{1}{2} {(\frac{{r}_{e}}{k})}^{\frac{-5}{2}}$$

$$C=\frac{5 \cdot A}{3\cdot \pi \cdot {Q}_{ext}}A=\frac{{C}_{w}}{\frac{4}{3}\cdot \pi \cdot {\rho }_{w}}$$

*k* is assumed to be 1.08, which relates r_e_ to the mean volume radius^25^. The extinction efficiency factor ($${Q}_{ext})$$ is approximated to be 2^25^, and $${\rho }_{w}$$ is the density of water. Values for C are calculated^35^, and the condensation rate ($${C}_{w}$$) is calculated using parcel model with ERA5 datasets as follows. The adiabatic liquid water content at different cloud base temperatures for a given 900 hPa is calculated using ERA5 data. The altitude at 900 hPa is defined as the cloud base pressure in this research. Then, the dependence of $${C}_{w}$$ on the cloud base temperature ($${T}_{c}$$) was obtained in order to perform a regression analysis between calculated water condensation and liquid water content. The maximum and minimum temperatures in the target domain are 265.8 (K) and 298.7 (K), thus the relationships within this temperature range are investigated. As a result,

$${C}_{W}=1.01{e}^{-4}{T}_{c}+5.90{e}^{-4}$$

between 265.8 (K) and 298.7 (K) was calculated which gives ${C}_{w}$ for each cloud base temperature. Daily data were used to calculate ${N}_{d}$. Some thresholds are used to obtain the low-level warm cloud as follows. (1) cloud phase(θ) is water (2) Cloud top pressure is above 680 hPa (3) Pixels whose $COD$ are the highest 90th percentiles^21^. Figure S33a shows the geographical distribution of calculated *N*_*d*_ in summer over the 33 year period from 1982 to 2014. The ${N}_{d}$ over the oceans close to North America and Europe are higher than that over any other regions by emitting anthropogenic aerosols. The probability of ${N}_{d}$ in the same region in summer over the 33 year period from 1982 to 2014 is investigated for verification of the calculation method (Fig. S33b). As a result, the mode ${N}_{d}$ value is 60 cm^−3^ and 70% of ${N}_{d}$ is calculated within the 150 cm^−3^, consistent with previous field observations^38,39^.

We evaluated the quality of the satellite data, particularly regarding the calculation of droplet number concentration (${N}_{d}$) as bellow. Mcgarragh et al.^18^, shows the relative uncertainty associated with cloud optical thickness and cloud particle size are both less than 20%. ${N}_{d}$ is proportional to 1/2 power of cloud optical thickness and − 5/2 power of cloud particle size (assuming that the uncertainties of these other variables are sufficiently small), so the relative uncertainty of ${N}_{d}$ is calculated as less than 60% ($<\frac{1}{2}\left(20\%\right)+\frac{5}{2}(20\%)$) ).

### Supplementary Information

Supplementary Information.

## Data Availability

The cloud data for this research is publicly available. CCI cloud data was obtained from the European Space Agency https://climate.esa.int/en/projects/cloud/data/. ERA5 data is available from the European Centre for Medium-Range Weather Forecasts at https://cds.climate.copernicus.eu/. The AOD provided by advanced very high resolution radiometer (AVHRR) was collected from the National Centers for Environmental Information https://www.ncei.noaa.gov/products/climate-data-records/avhrr-aerosol-optical-thickness. The ice core data is available at https://eprints.lib.hokudai.ac.jp/dspace/handle/2115/67127. Code for calculations and data processing are available from the corresponding author upon request.
