# Supplementary material for: Long-term relationships between summer clouds and aerosols over mid-high latitudes of the Northern Hemisphere
Source: Sci Rep. 2024 Apr 20;14:9059. doi: 10.1038/s41598-024-59817-7 (PMC11032361; doi:10.1038/s41598-024-59817-7)
Supplement: Supplementary file 1 — Supplementary Information. [file 41598_2024_59817_MOESM1_ESM.docx]

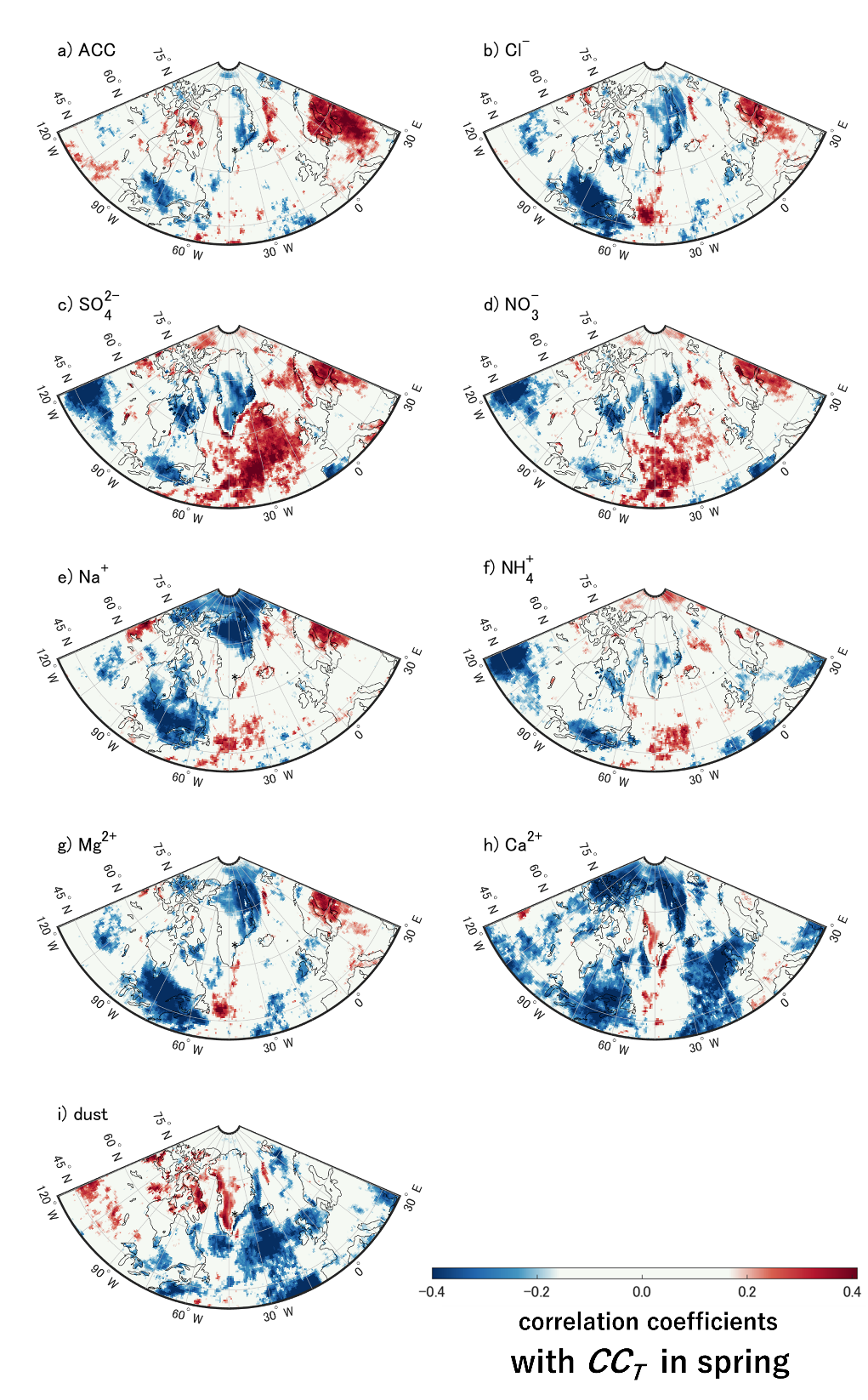


Figure S1. Geographical distributions of correlation coefficient between aerosol proxies; a) accumulation rate, b) Cl^–^, c) SO_4_^2–^, d) NO_3_^–^, e) Na^+^, f) NH_4_^+^, g) Mg^2+^, h) Ca^2+^, and i) dust concentration, against cloud amounts of total level (${CC}_{T}$) in spring over the 33-year period from 1982 to 2014. Asterisk denotes the southeast Dome of Greenland where the ice core was drilled^13^


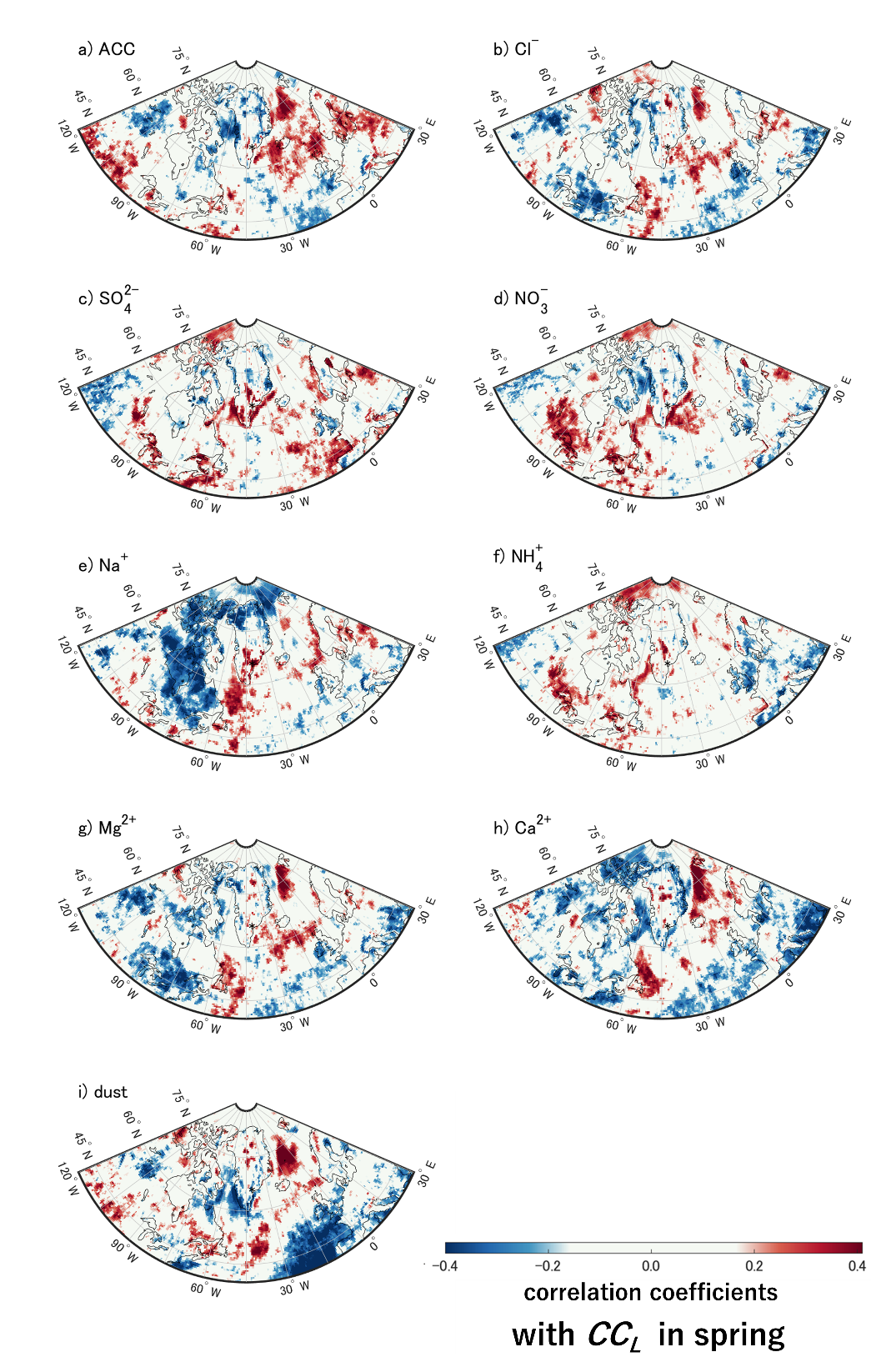


Figure S2. As Fig. S1, but for cloud amounts of low level (${CC}_{L}$) in spring.


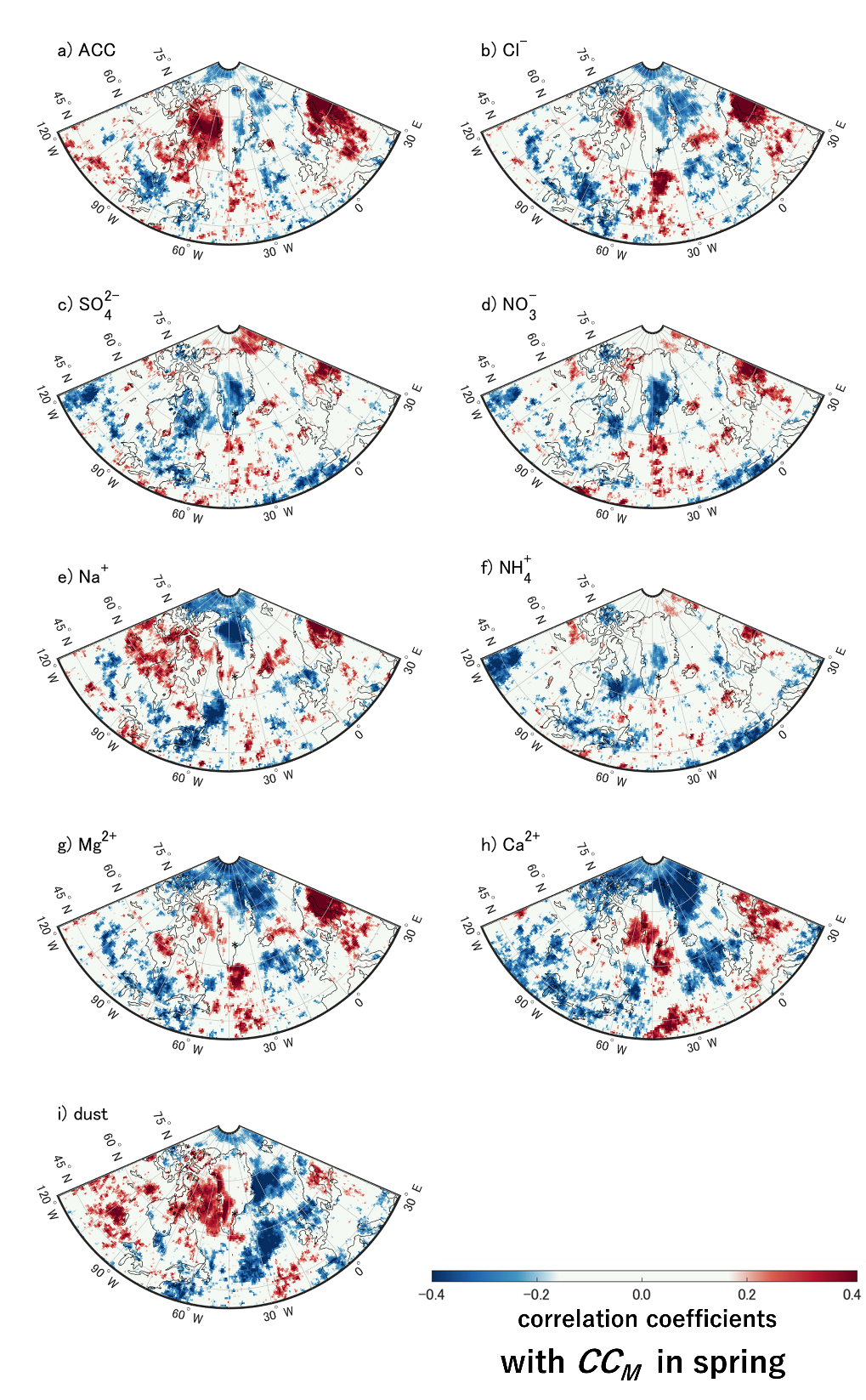


Figure S3. As Fig. S1, but for cloud amounts of middle level (${CC}_{M}$) in spring.


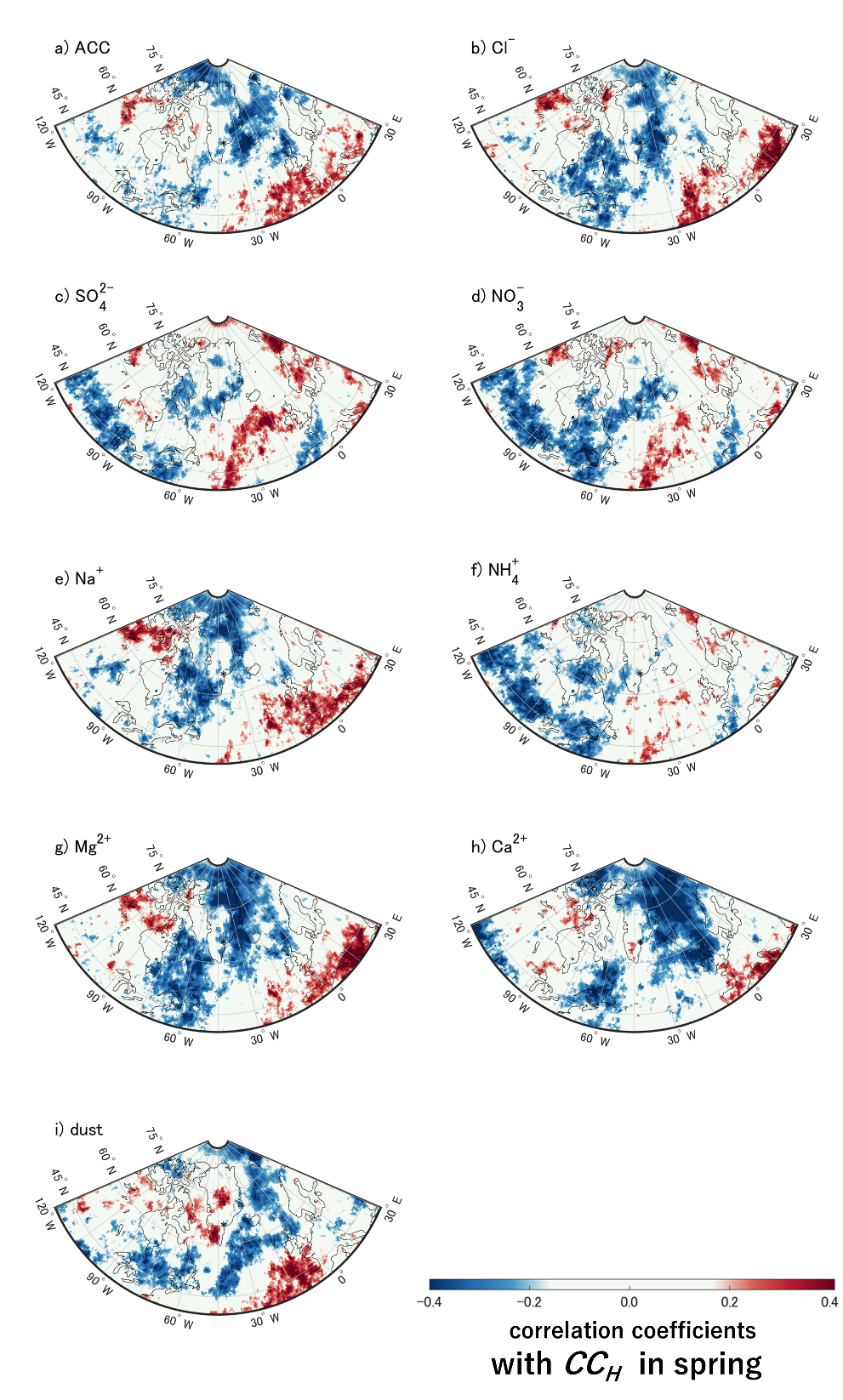


Figure S4. As Fig. S1, but for cloud amounts of high level (${CC}_{H}$) in spring.


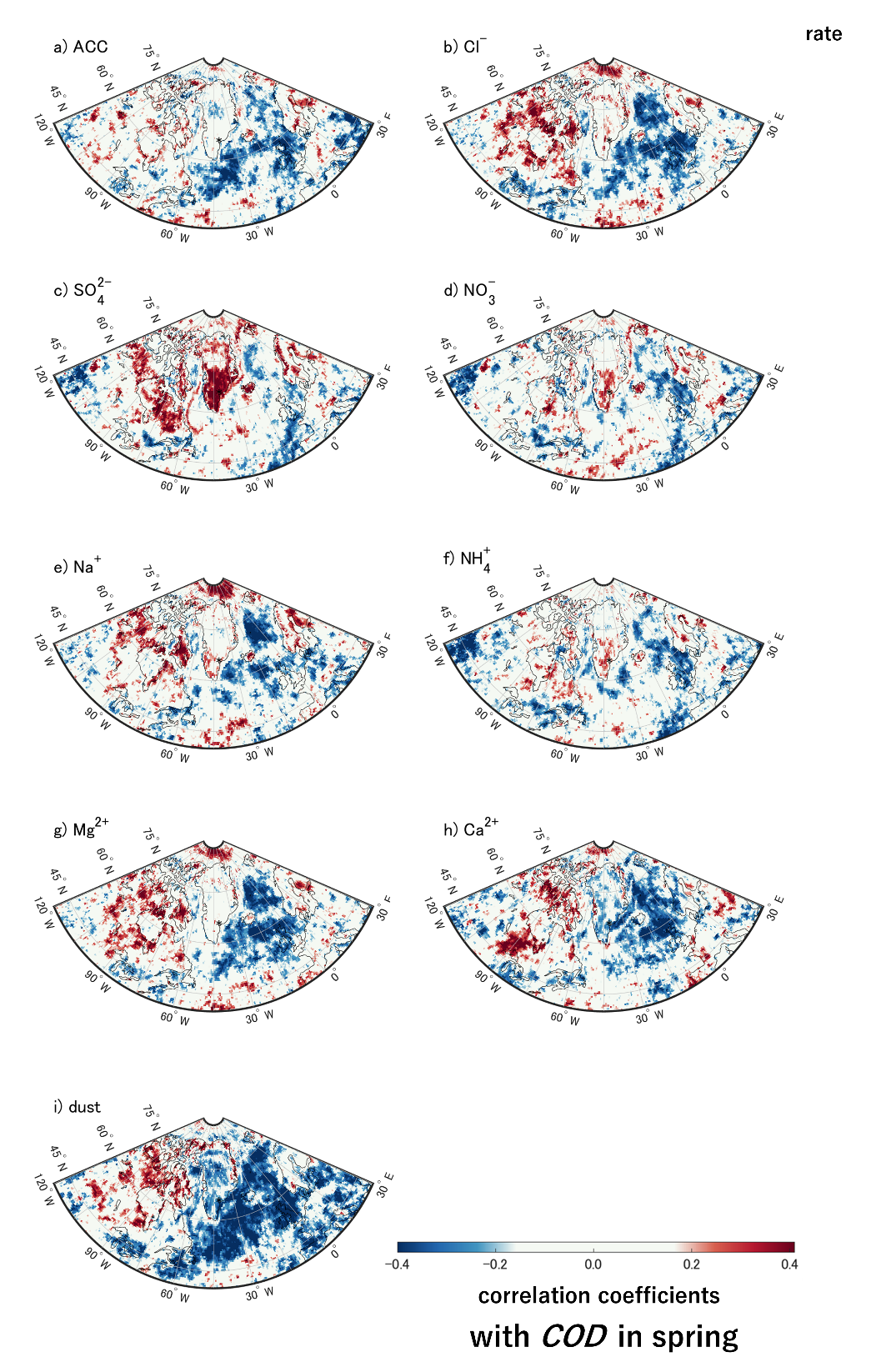


Figure S5. As Fig. S1, but for cloud optical depth ($COD$) in spring.


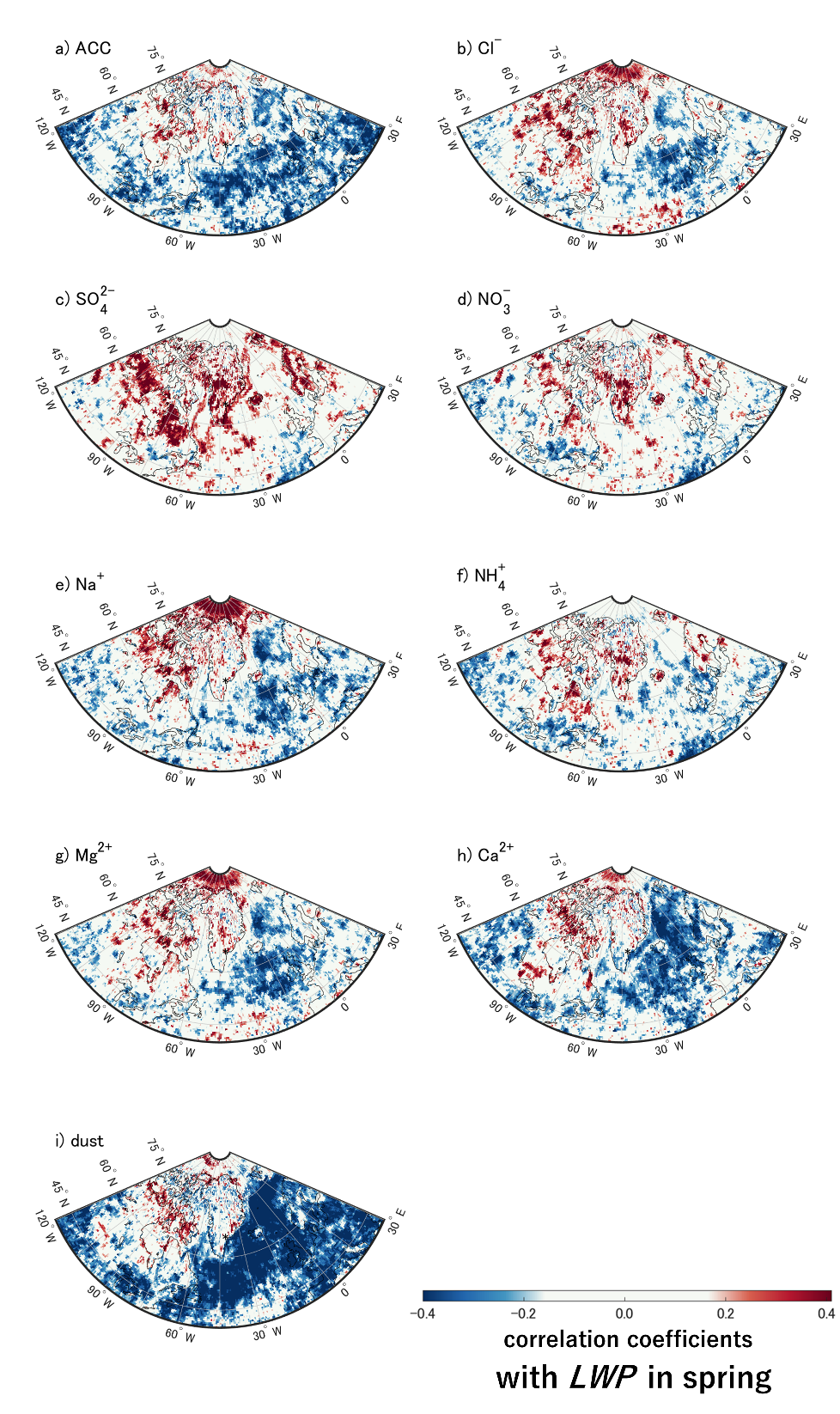


Figure S6. As Fig. S1, but for liquid water path ($LWP$) in spring.

**
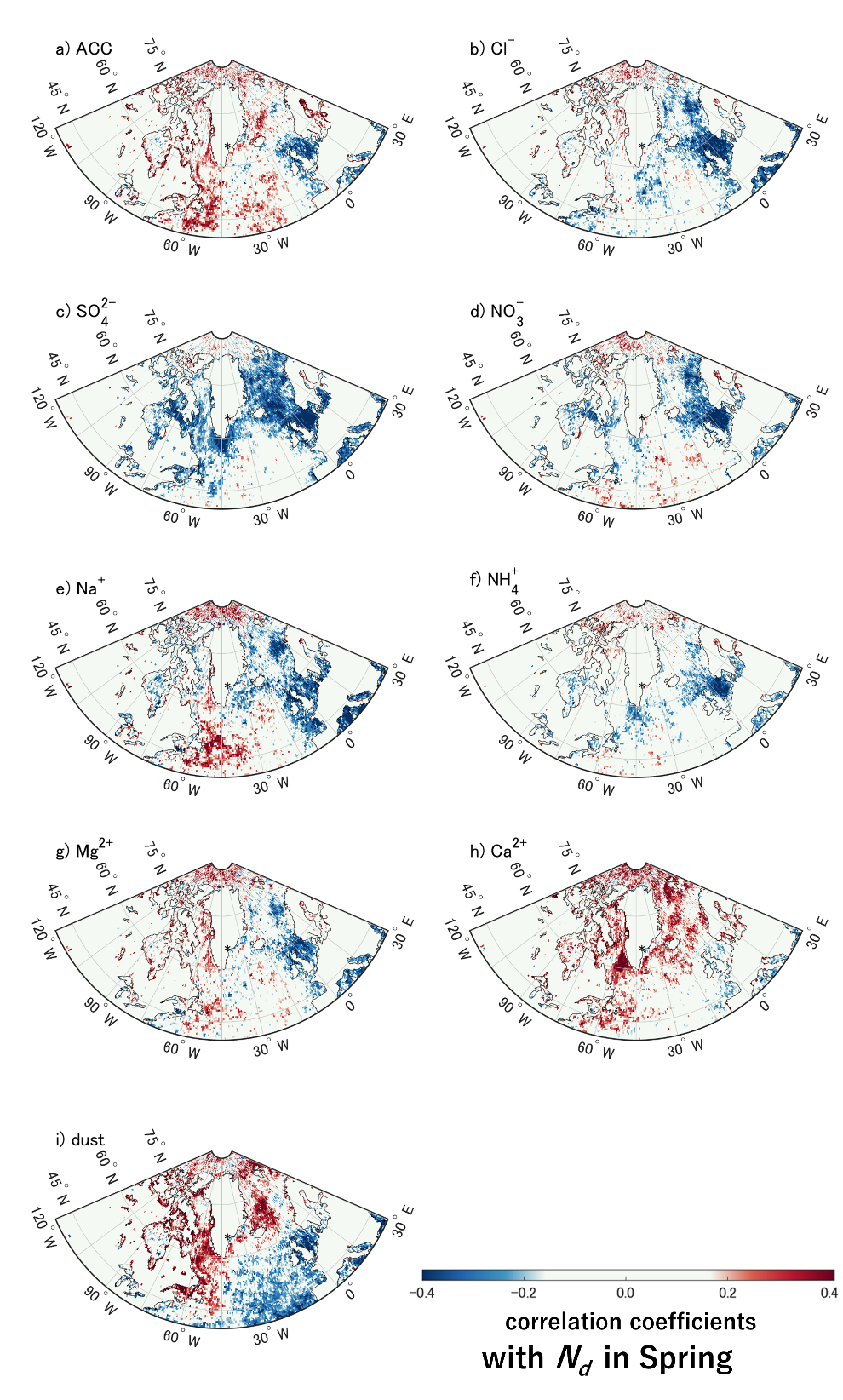
**

Figure S7. As Fig. S1, but for cloud droplet concentration ($N_{d}$) in spring.


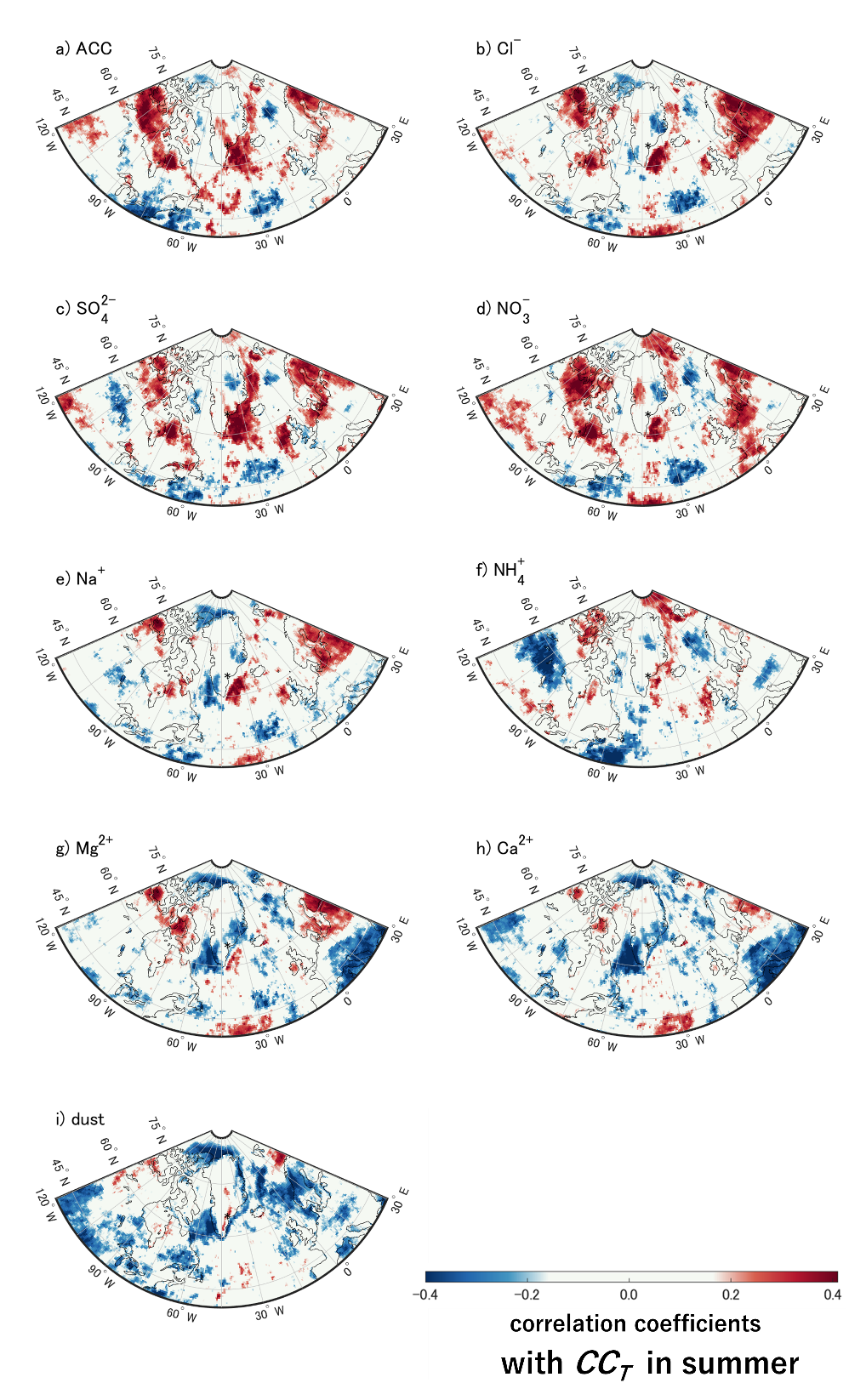


Figure S8. As Fig. S1, but for summer.


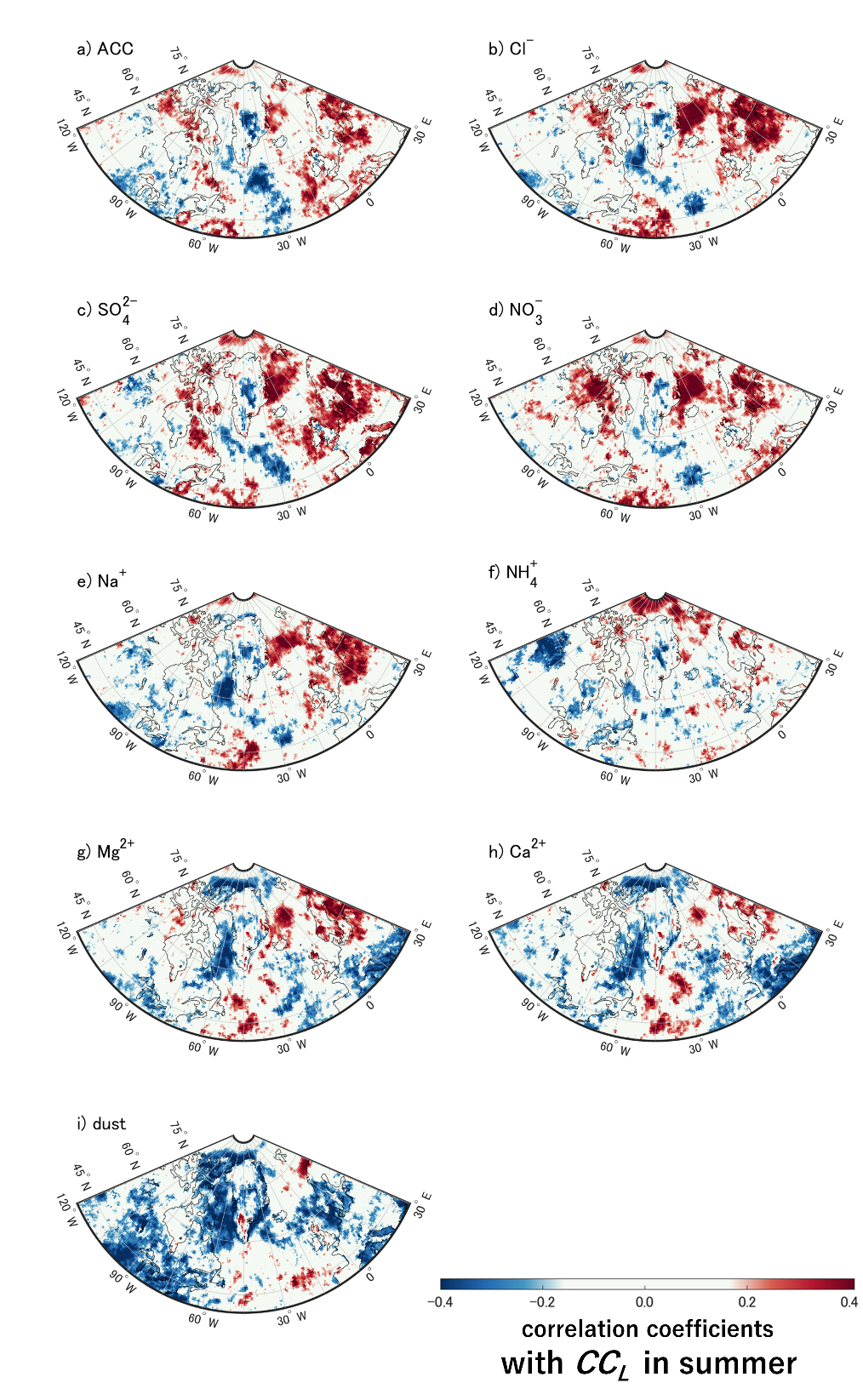


Figure S9. As Fig. S2, but for summer.


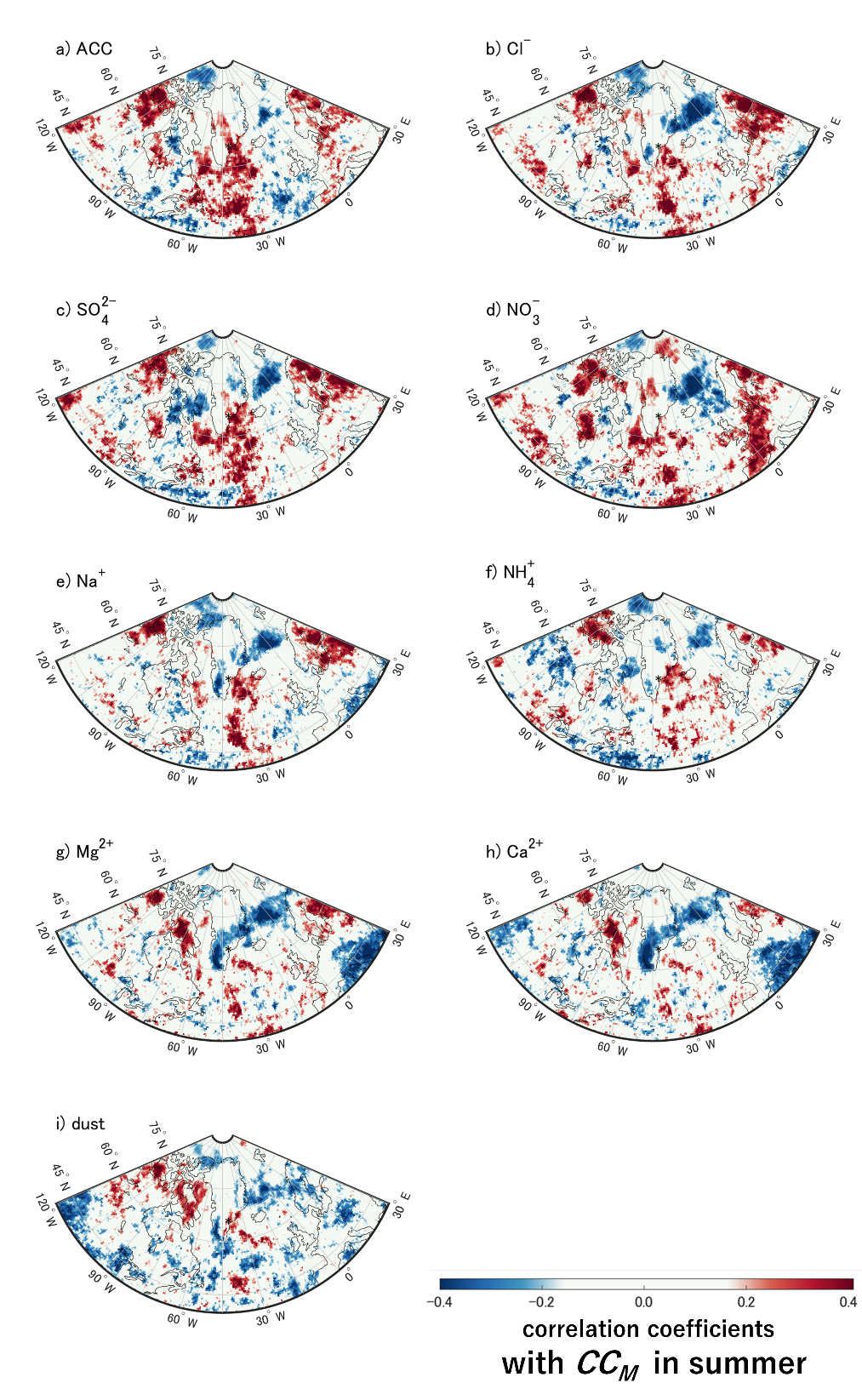


Figure S10. As Fig. S3, but for summer.


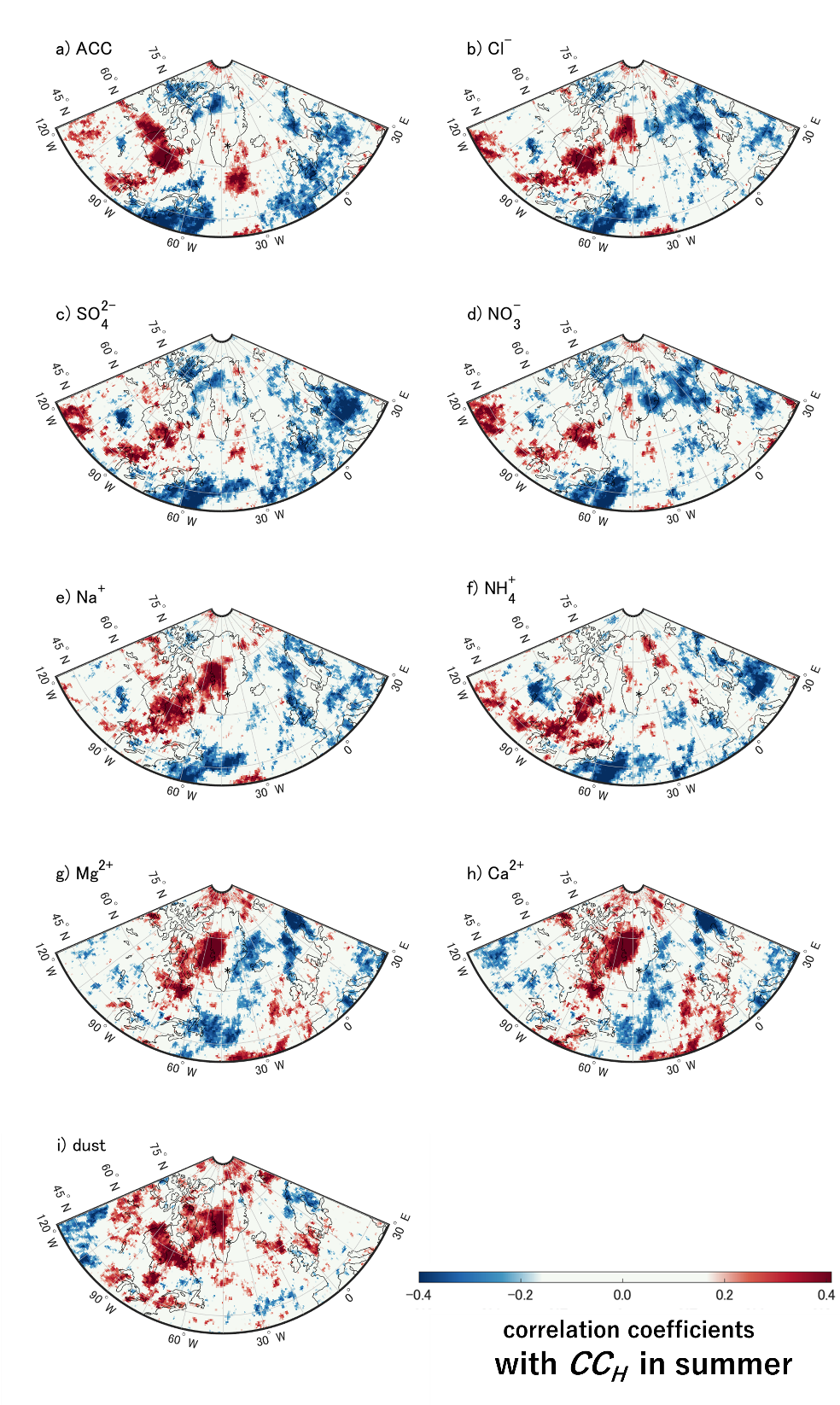


Figure S11. As Fig. S4, but for summer.


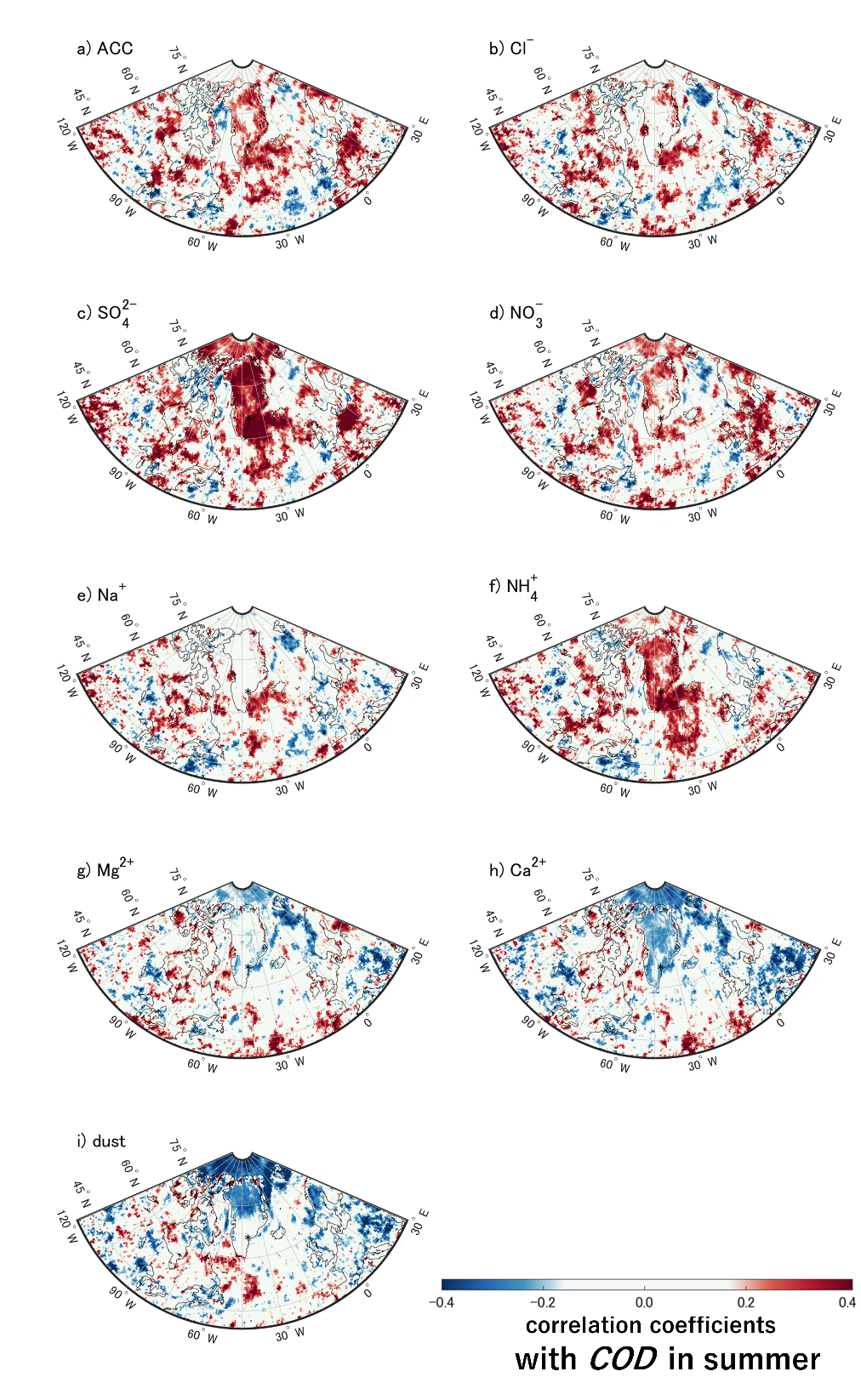


Figure S12. As Fig. S5, but for summer.


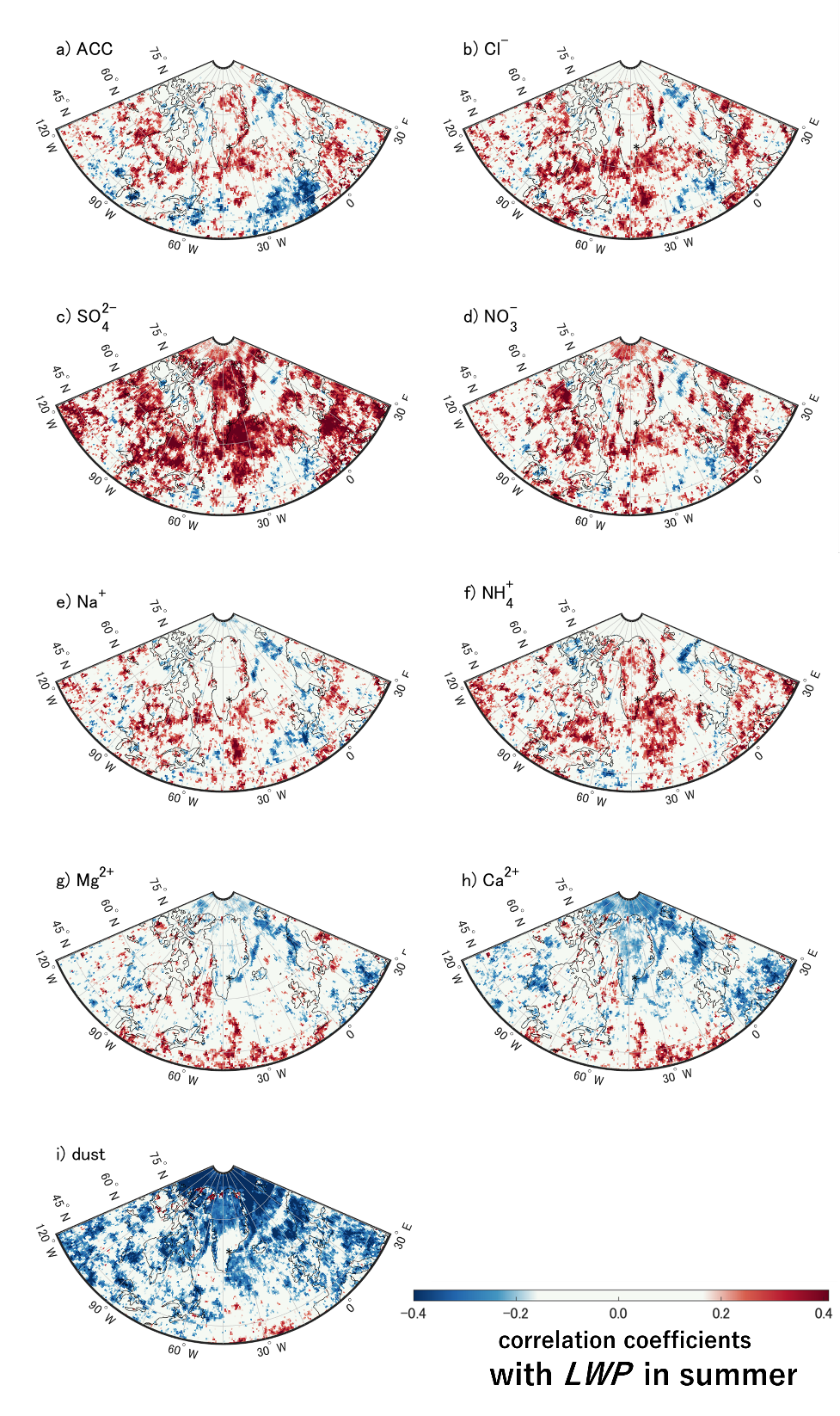


Figure S13. As Fig. S6, but summer.

**
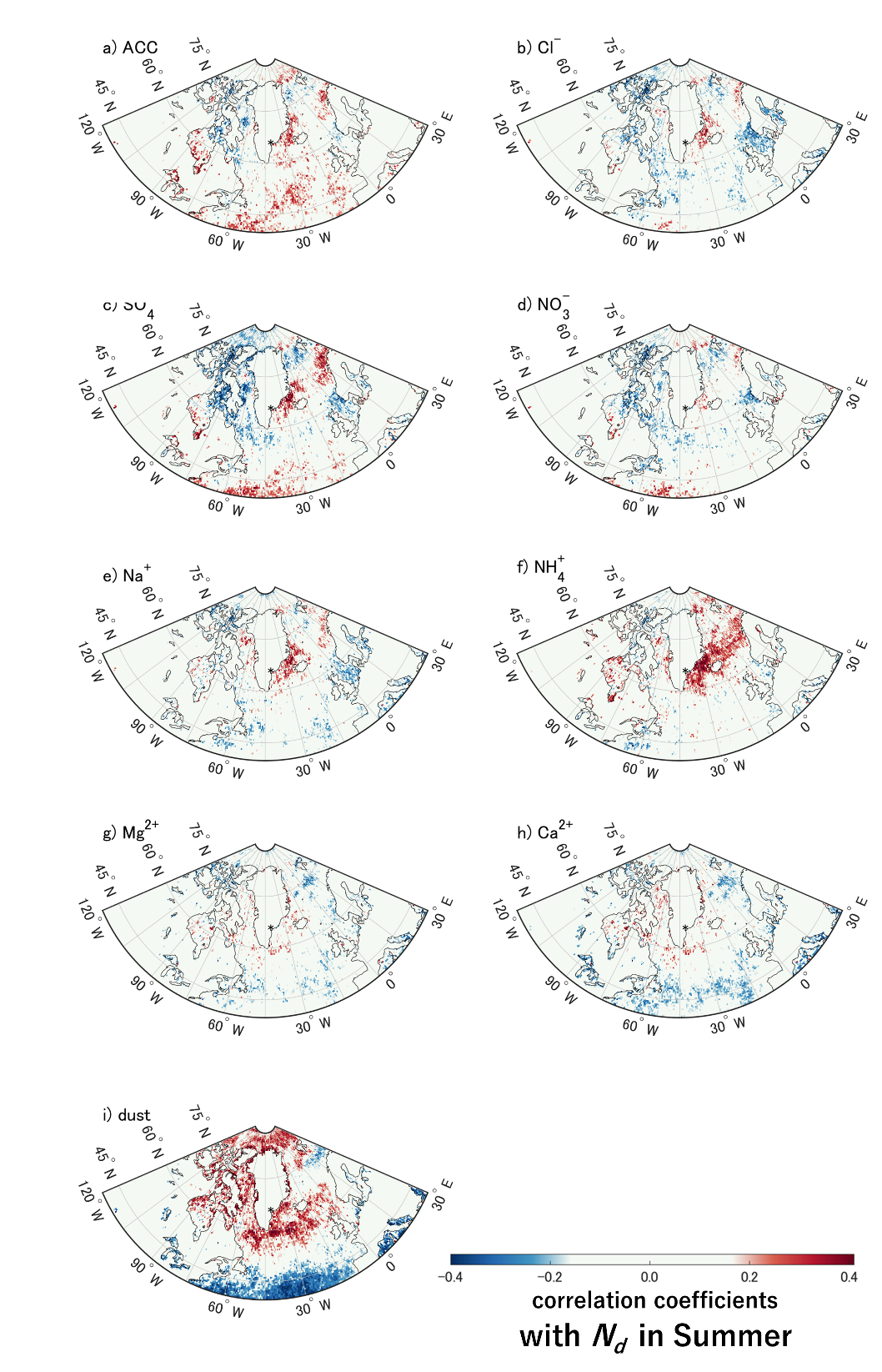
**

Figure S14. As Fig. S7, but for summer.

**
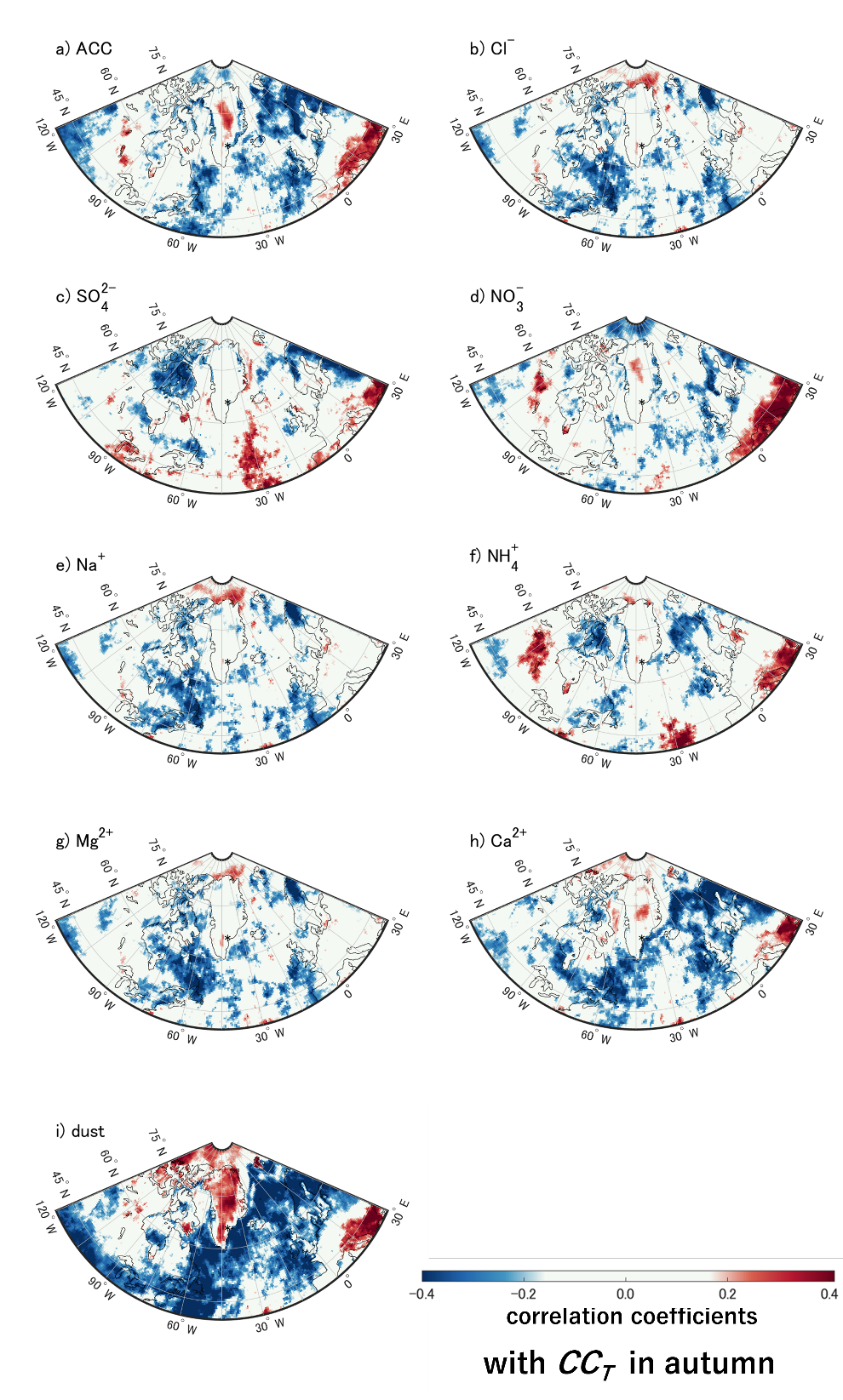
**

Figure S15. As Fig. S1, but for autumn.

**
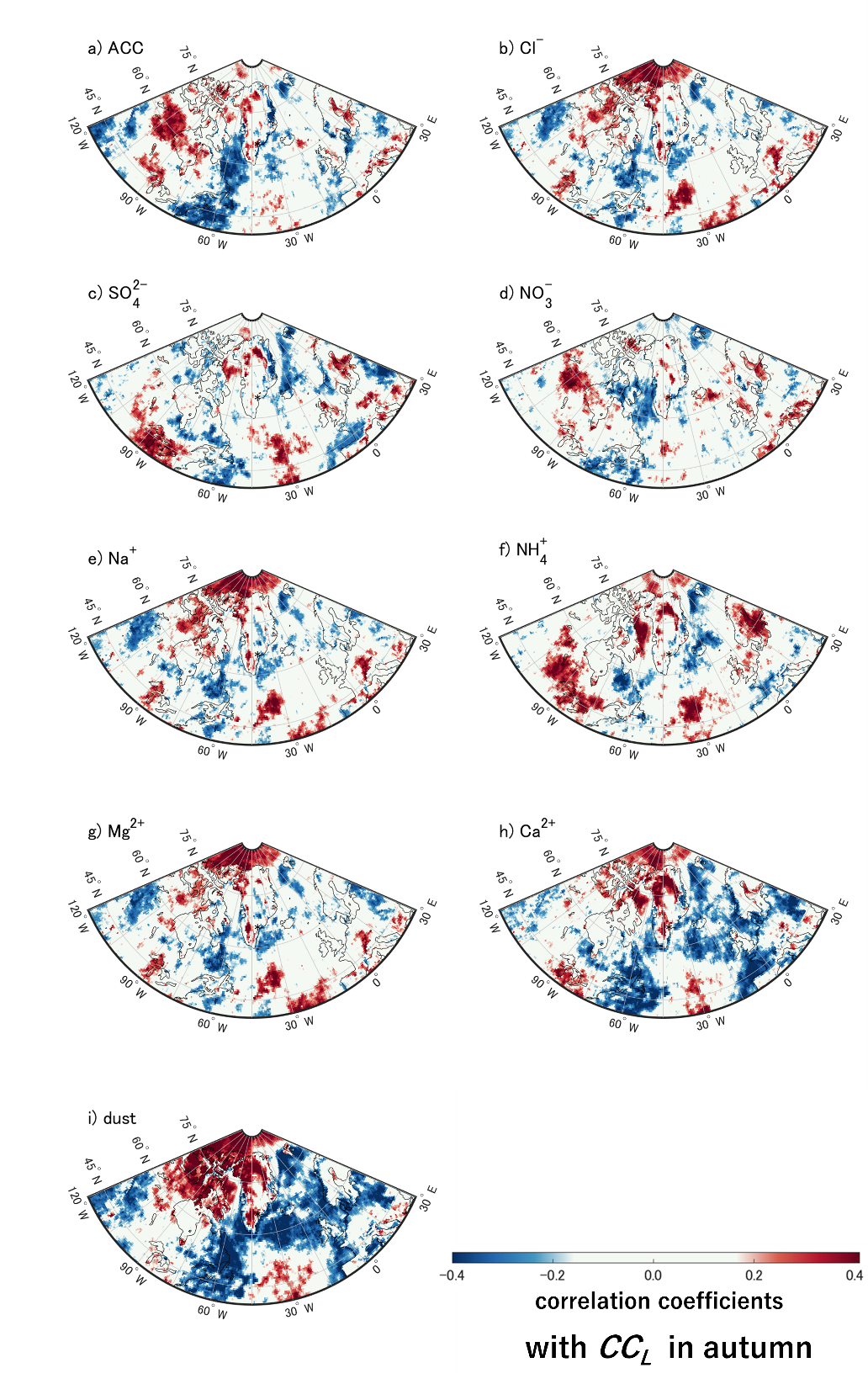
**

Figure S16. As Fig. S2, but for autumn.

**
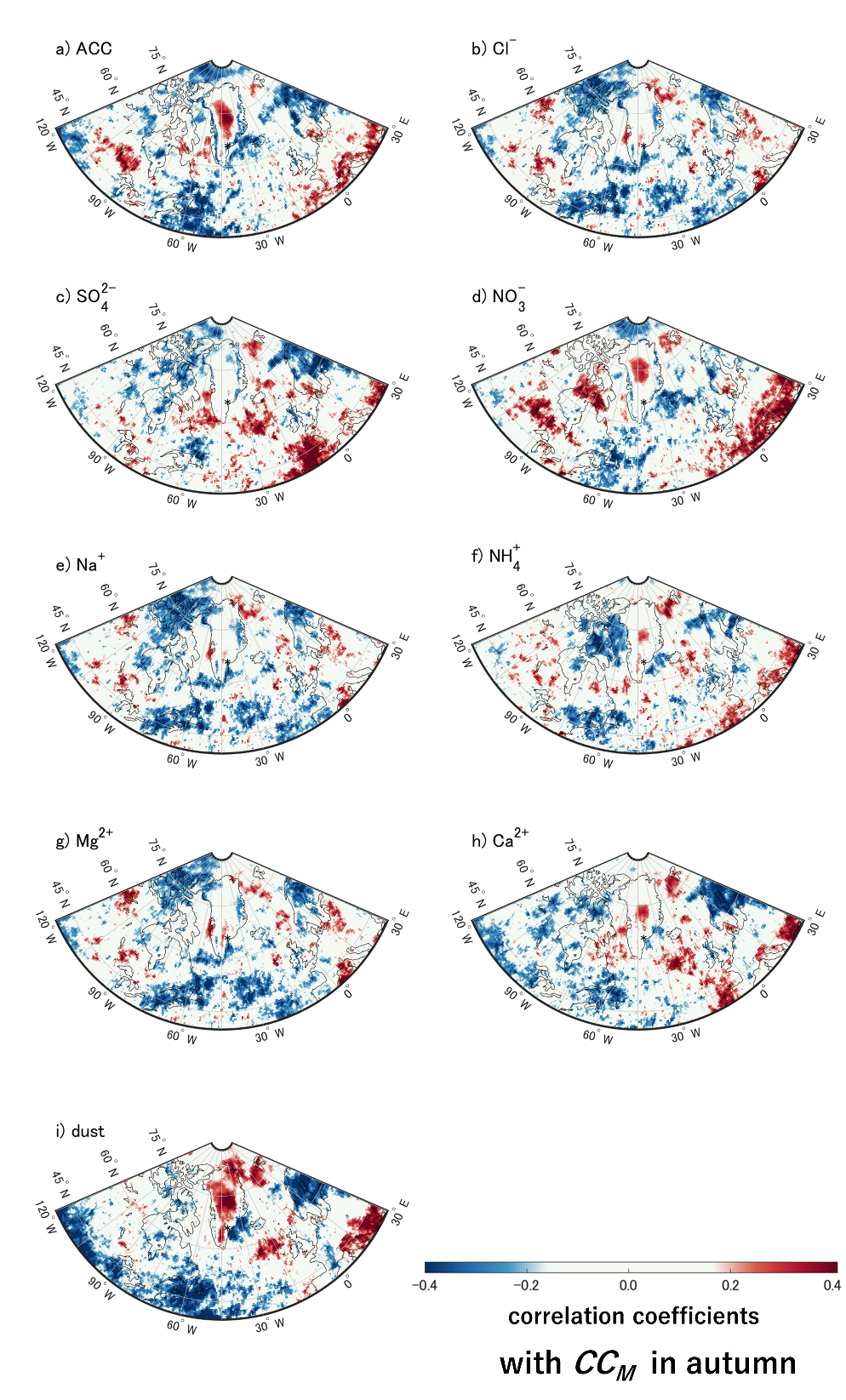
**

Figure S17. As Fig. S3, but for autumn.

**
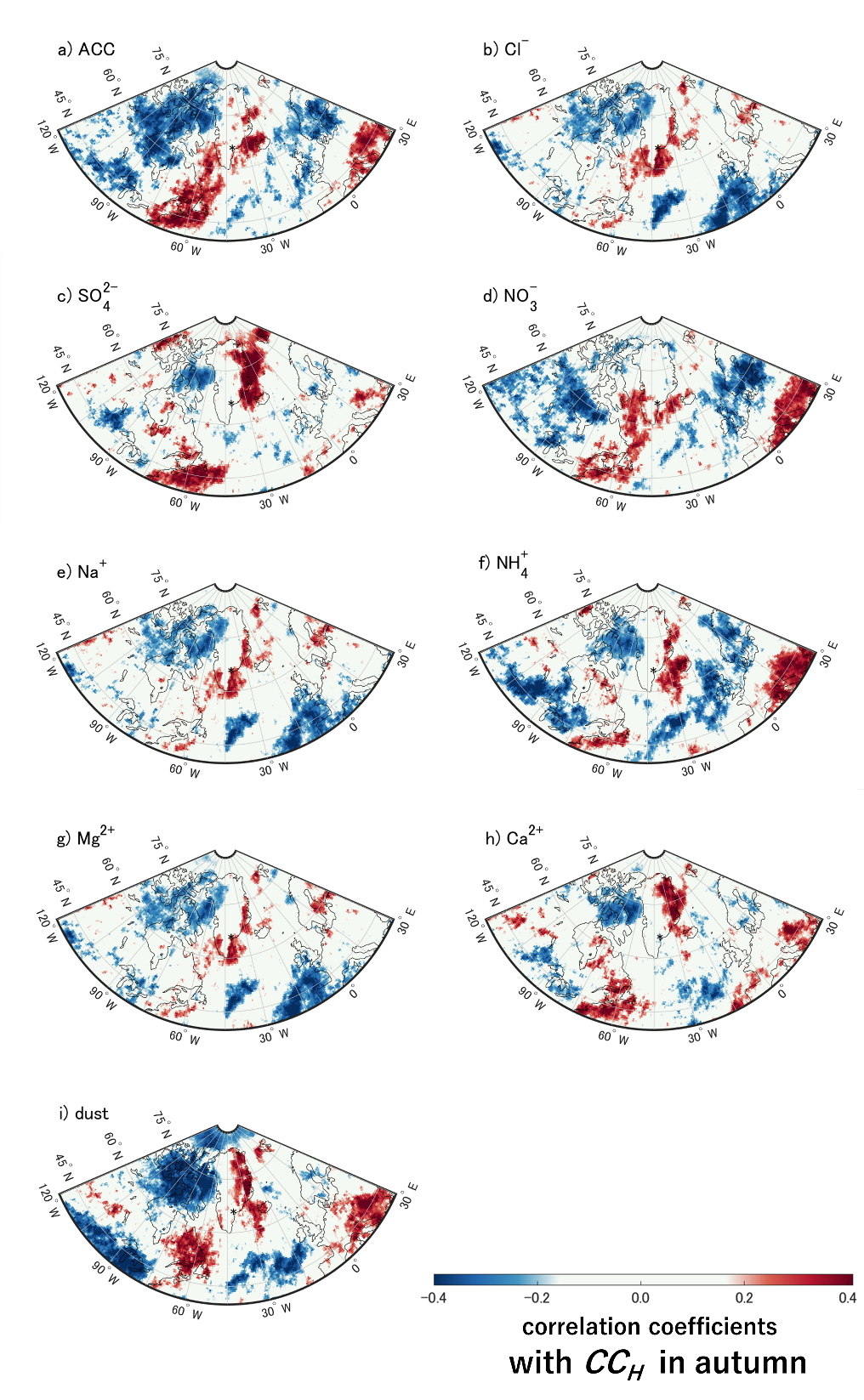
**

Figure S18. As Fig. S4, but for autumn.

**
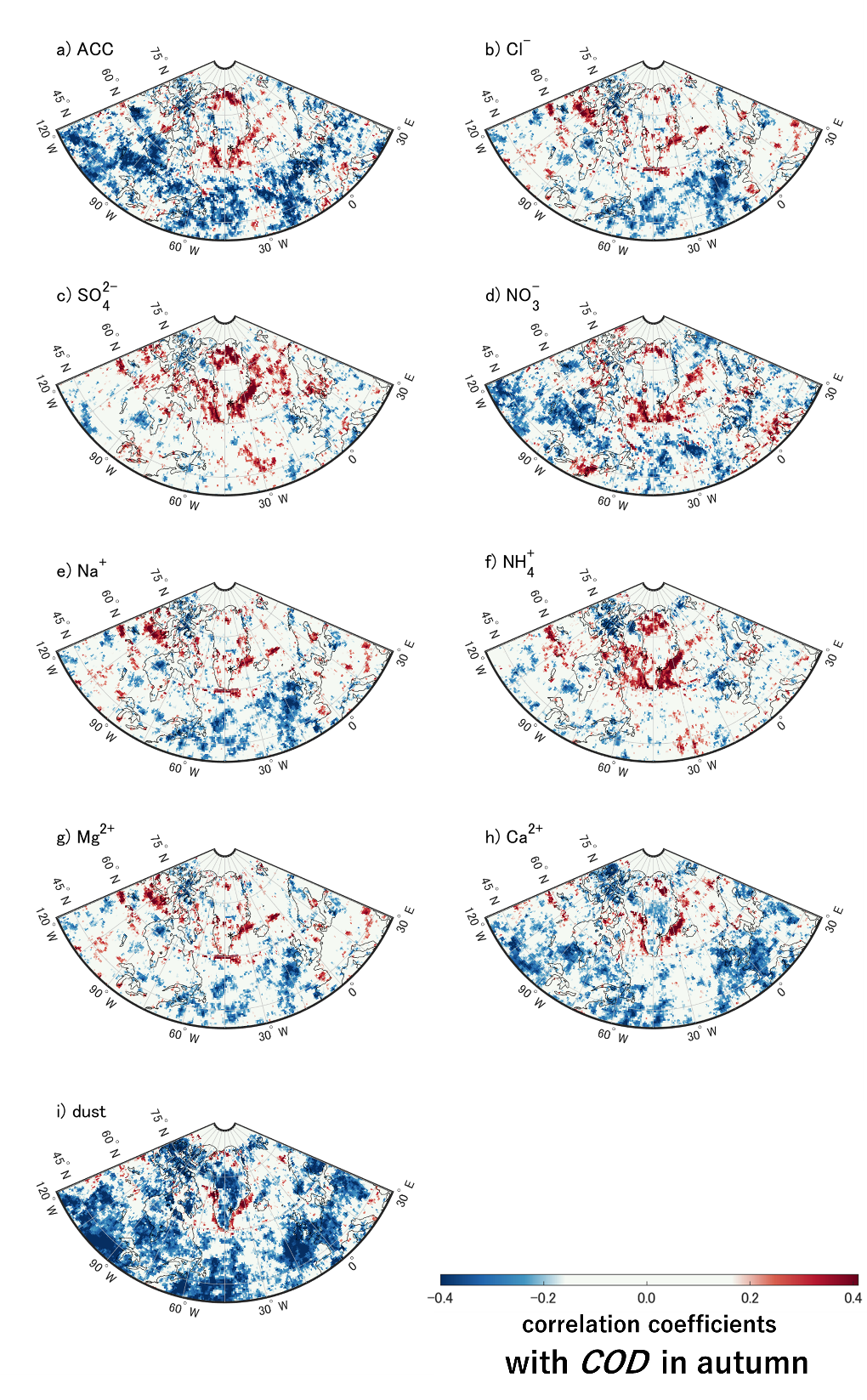
**

Figure S19. As Fig. S5, but for autumn.

**
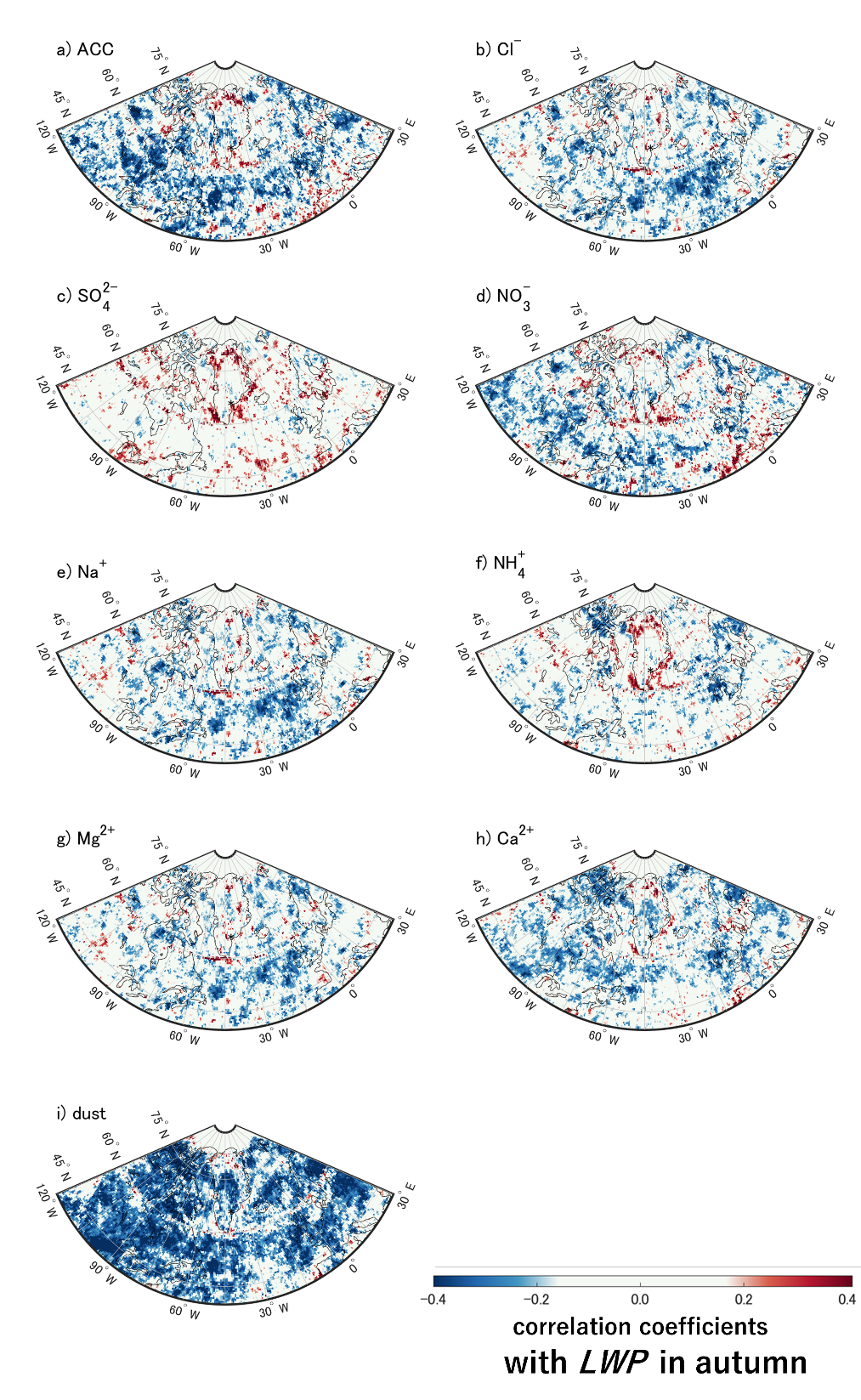
**

Figure S20. As Fig. S6, but for autumn.

**
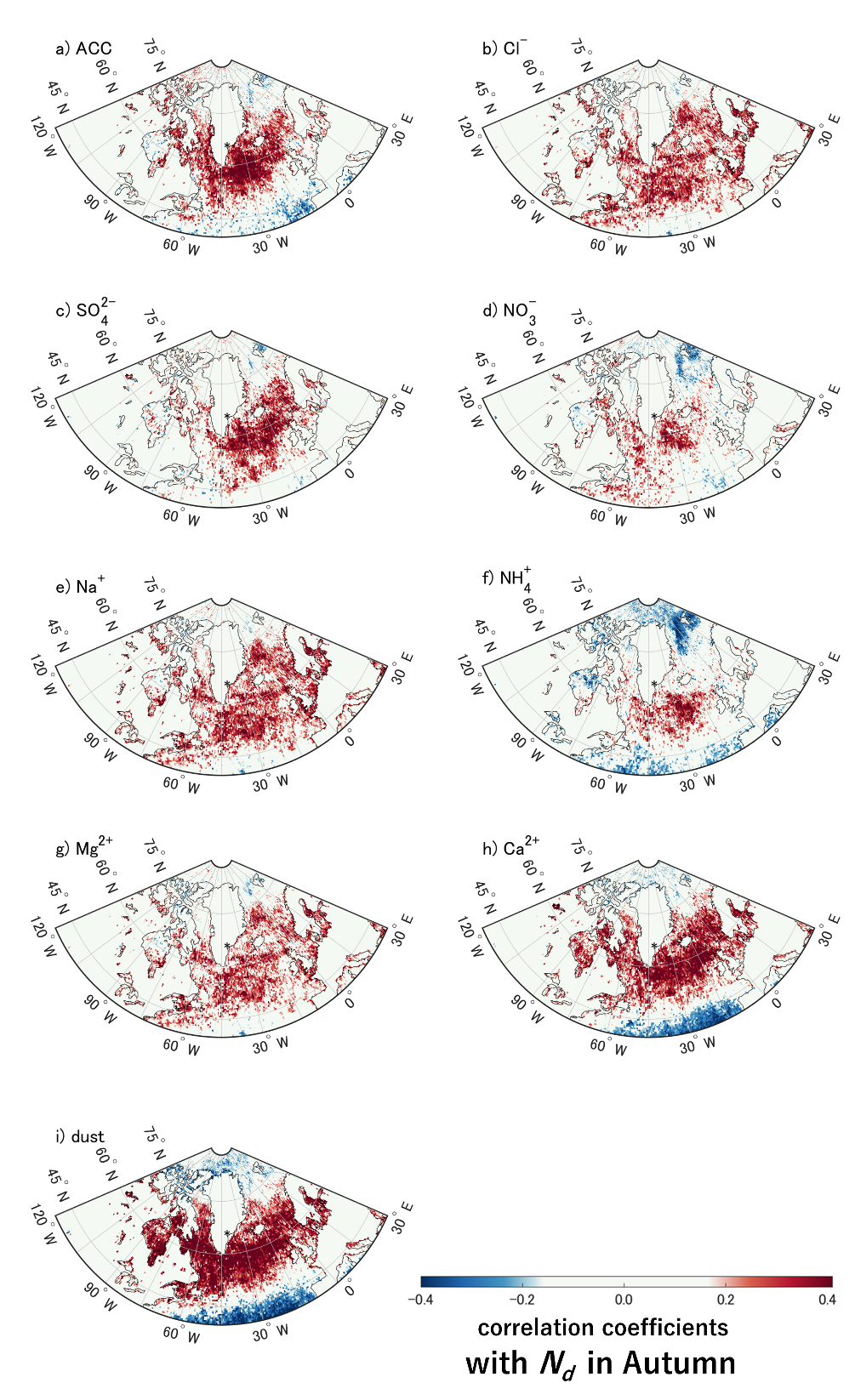
**

Figure S21. As Fig. S7, but for autumn.

**
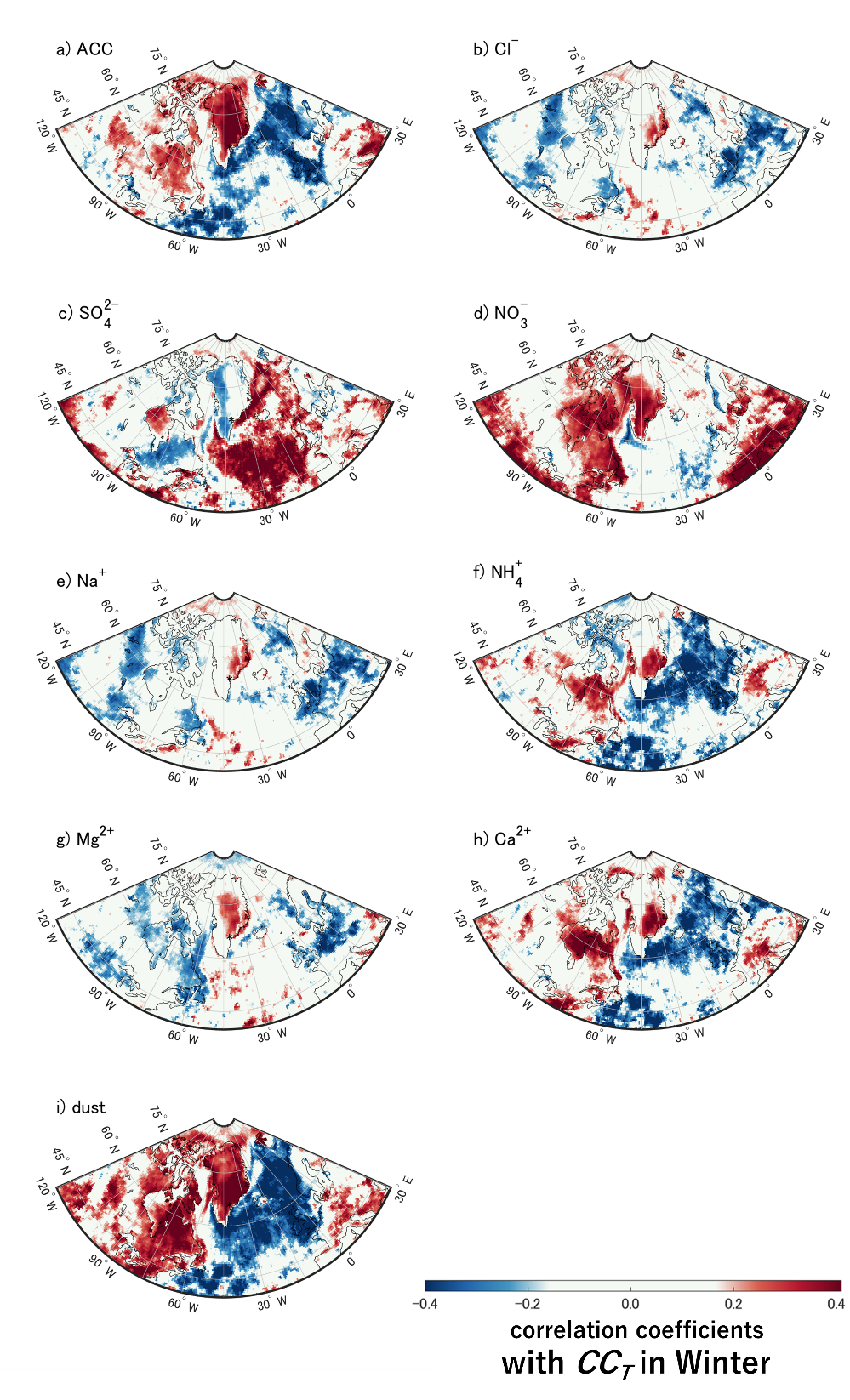
**

Figure S22. As Fig. S1, but for winter.

**
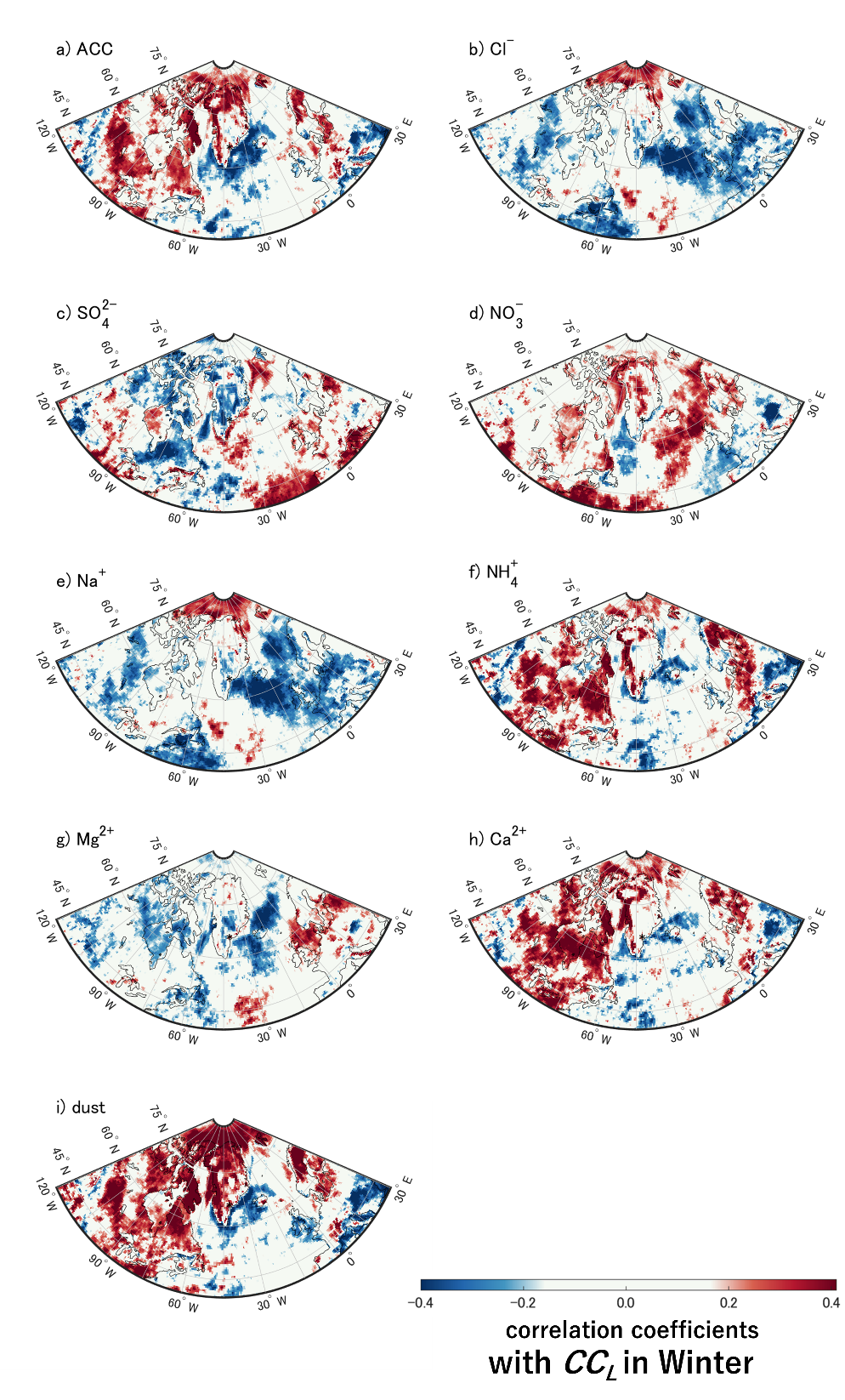
**

Figure S23. As Fig. S2, but for winter.


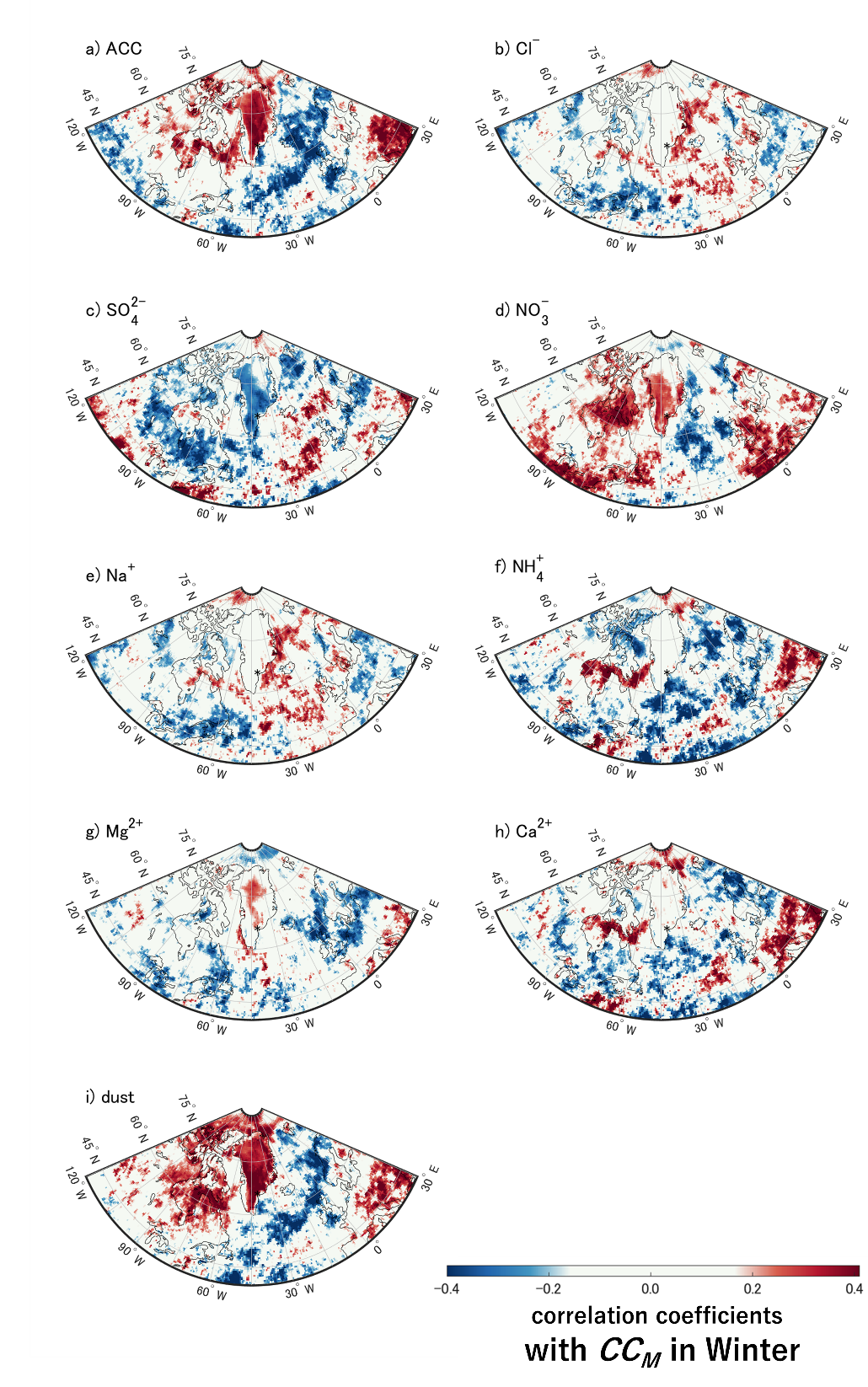


Figure S24. As Fig. S1, but for winter.


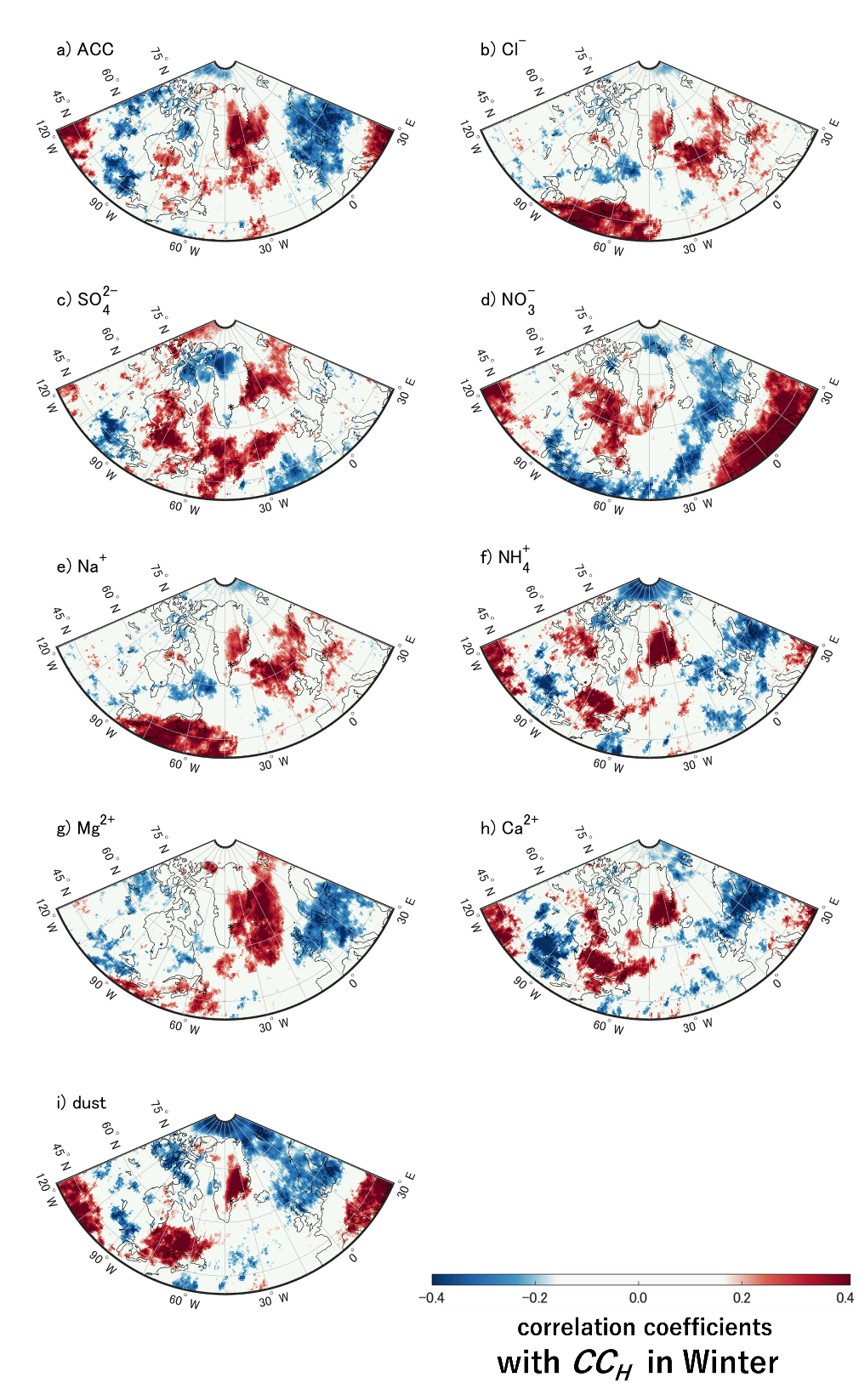


Figure S25. As Fig. S1, but for winter.


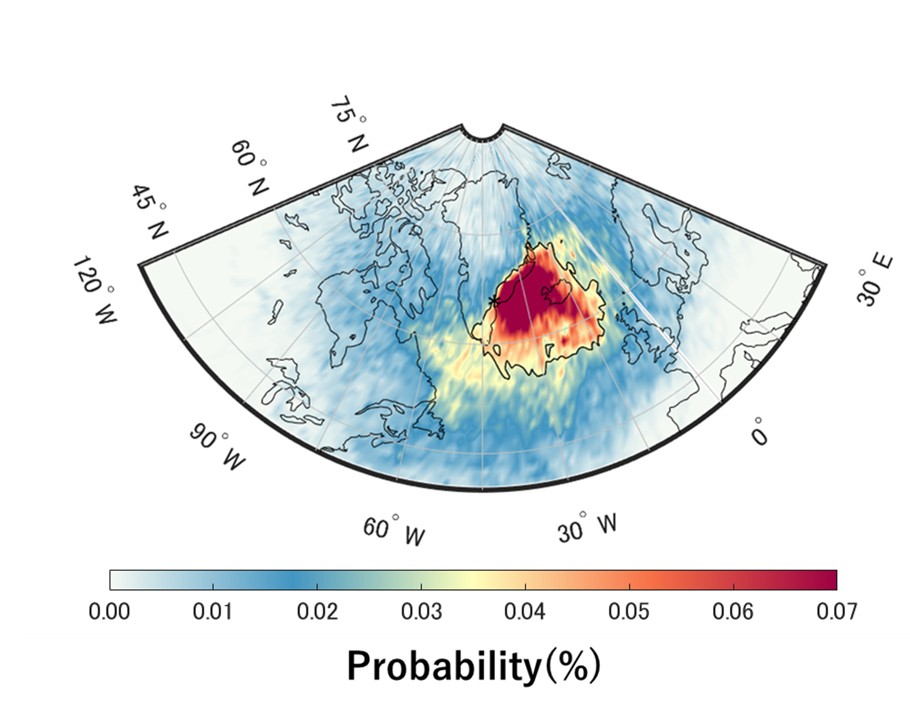


Figure S26. The probability distribution of an air mass arriving at the SE-Dome site (asterisk) from a 14-days 3-D backward trajectory analysis with a resolution of 1º for the summer season from 1982 to 2014. The air mass path is constrained under 1,500 m above ground level. Blackline shows the area where the integrated probability of air mass to the SE-Dome site was more than 50%.


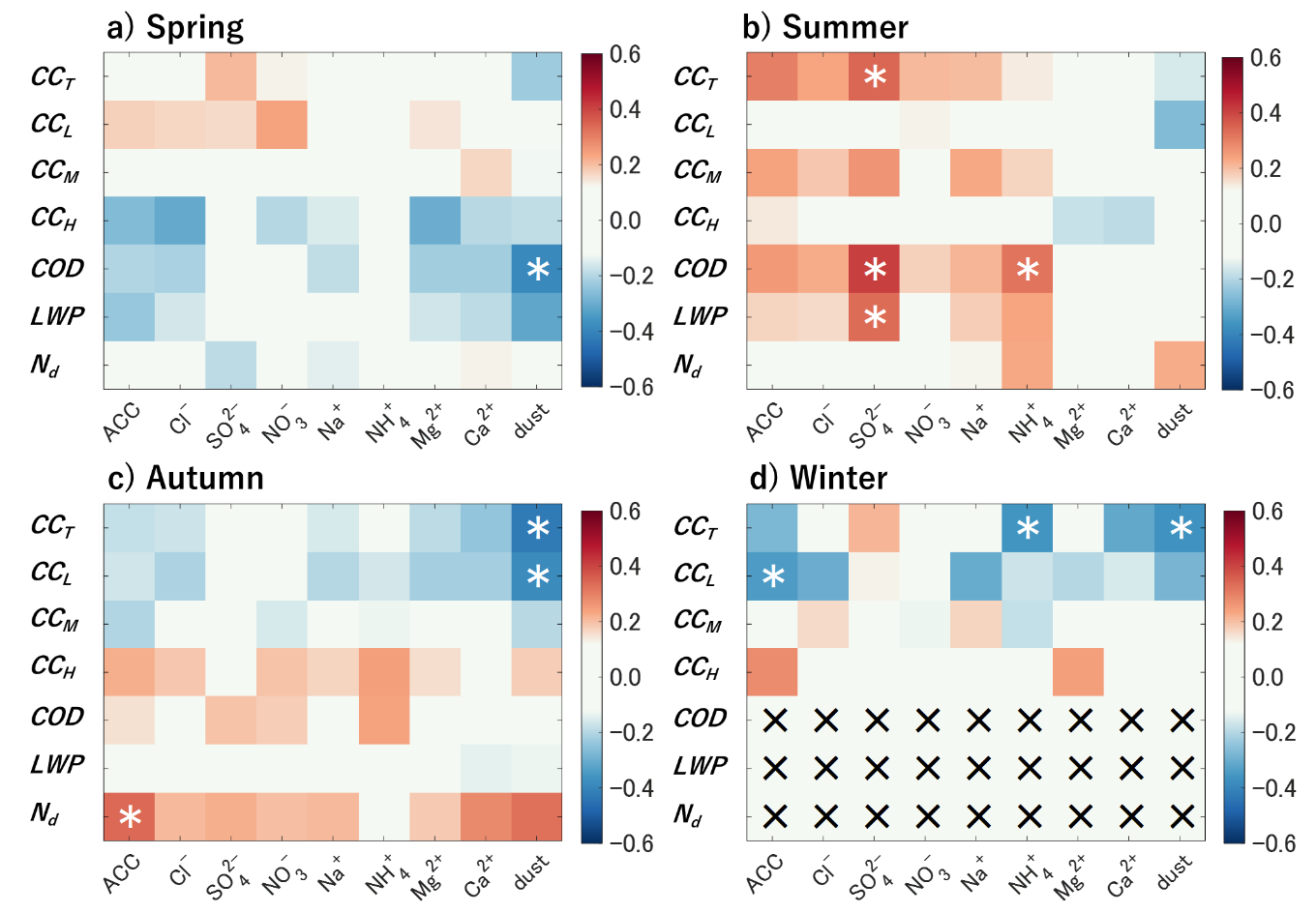


Figure S27. area-averaged correlation coefficients between aerosol proxies preserved in the SE-Dome ice core and cloud properties in the target domain in **a,** spring, **b,** summer, **c,** autumn, and **d,** winter from 1982 to 2014. The vertical axis shows the cloud properties; cloud amounts of total level (${CC}_{T}$), low level (${CC}_{L}$), middle level (${CC}_{M}$), high level (${CC}_{H}$), cloud optical depth ($COD$), liquid water path ($LWP$), and cloud droplet concentration ($N_{d}$), respectively. The horizontal axis shows the aerosol proxies preserved in the SE-Dome ice core. White asterisks denotes *p* < 0.05. Crosses in panel (d) denote “data not available” due to the polar night.


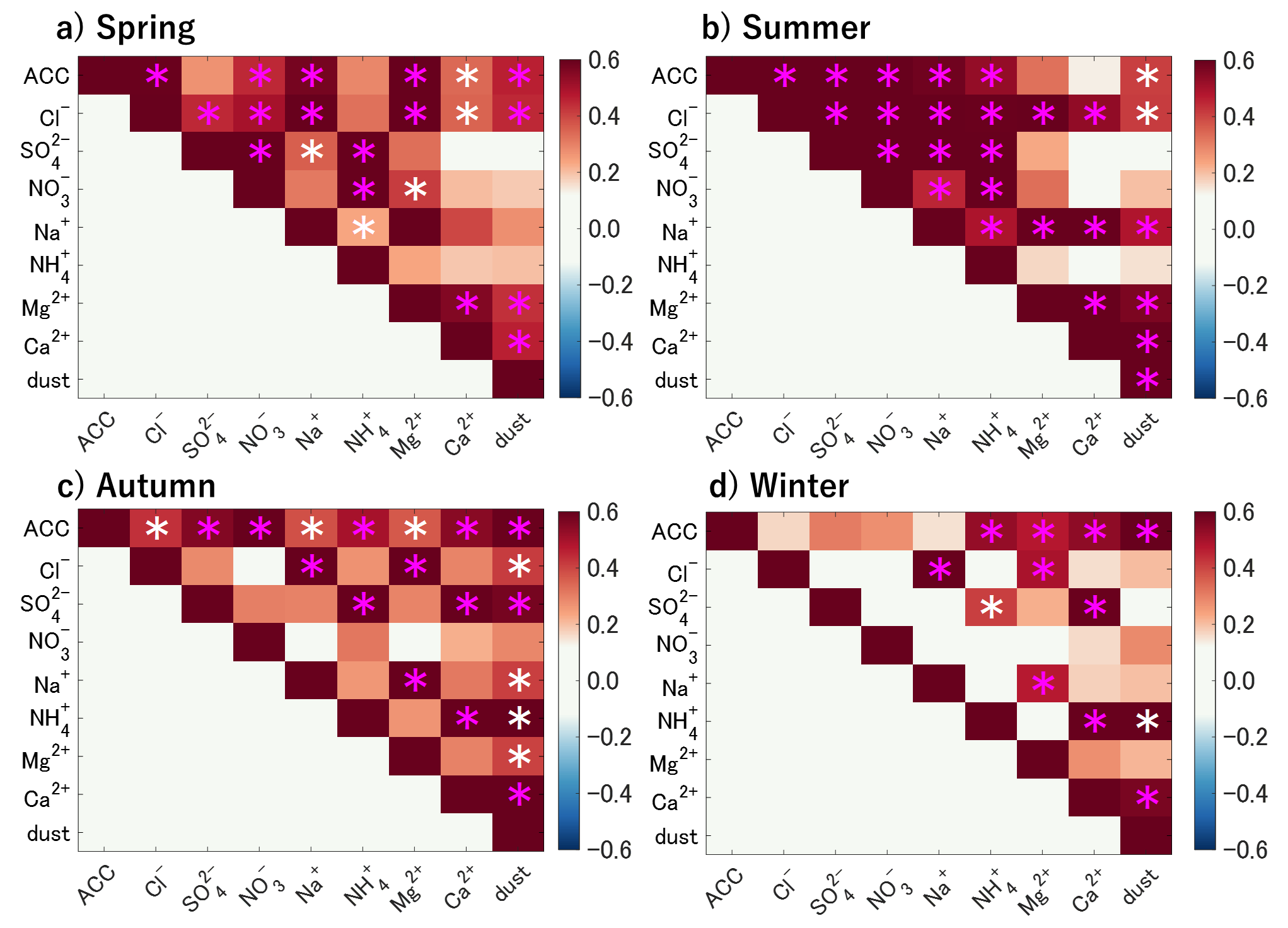


Figure S28. **C**orrelation coefficients among the aerosol proxies preserved in the SE-Dome ice core in **a,** spring, **b,** summer, **c,** autumn, and **d,** winter from 1982 to 2014, respectively. White and magenta asterisks denote *p* < 0.05 and *p* < 0.01, respectively.


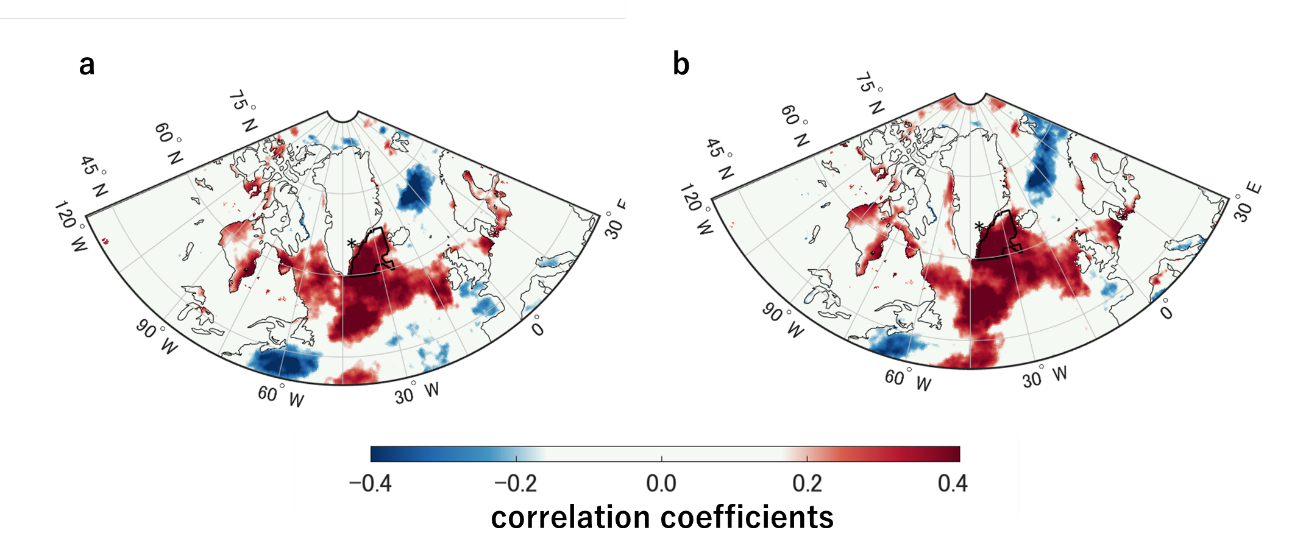


Figure S29. Geographical distributions of correlation coefficient between the SO_4_^2-^flux and **a,** ${CC}_{L}$ and **b,** ${CC}_{T}$ in the ERA5 reanalysis dataset with a resolution of 0.5º in summer from 1982 to 2014. Blackline denotes the target domain in this research.


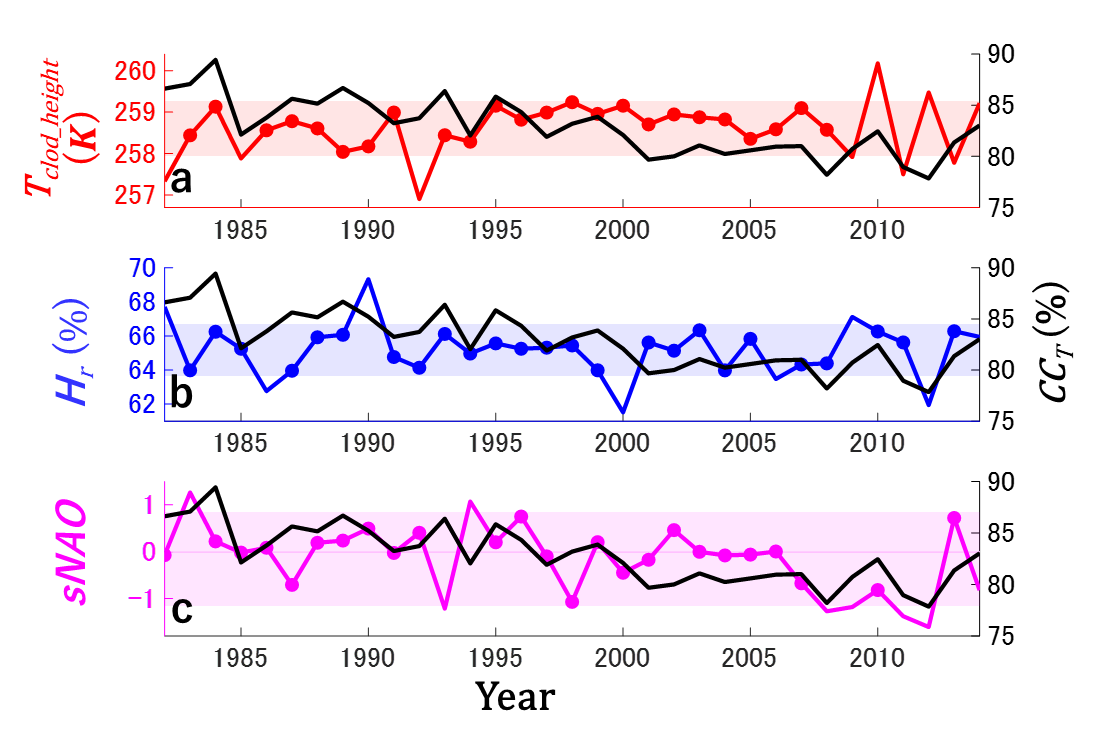


Figure S30. Temporal changes in summer mean **a,** *T_2m_*, **b,** *H_r_* , and ***c,*** *sNAO* in the target domain (left axes). *CC_T_* (black lines, right axes) is also shown in all panels. Light shaded areas denote ±1 standard deviation for the entire period. The period with dots were within the ±1 standard deviation.


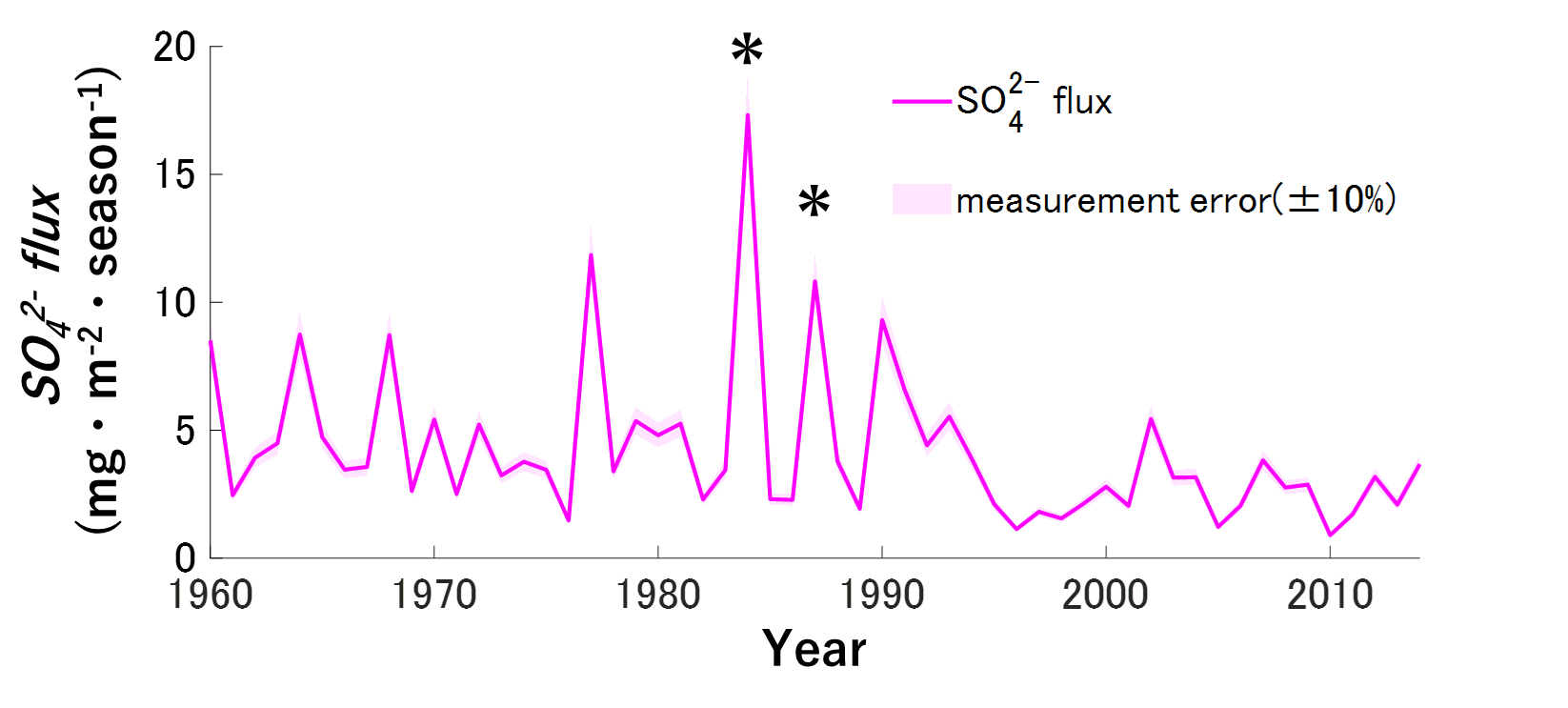


Figure S31. The SO_4_^2-^ flux in summer over the 55-year period from 1960 to 2014 in SE-Dome ice core. The shaded area means the measurement error^13^. Asterisks means the period higher than 2 standard deviations from 1982 to 2014 (1984 and 1987).


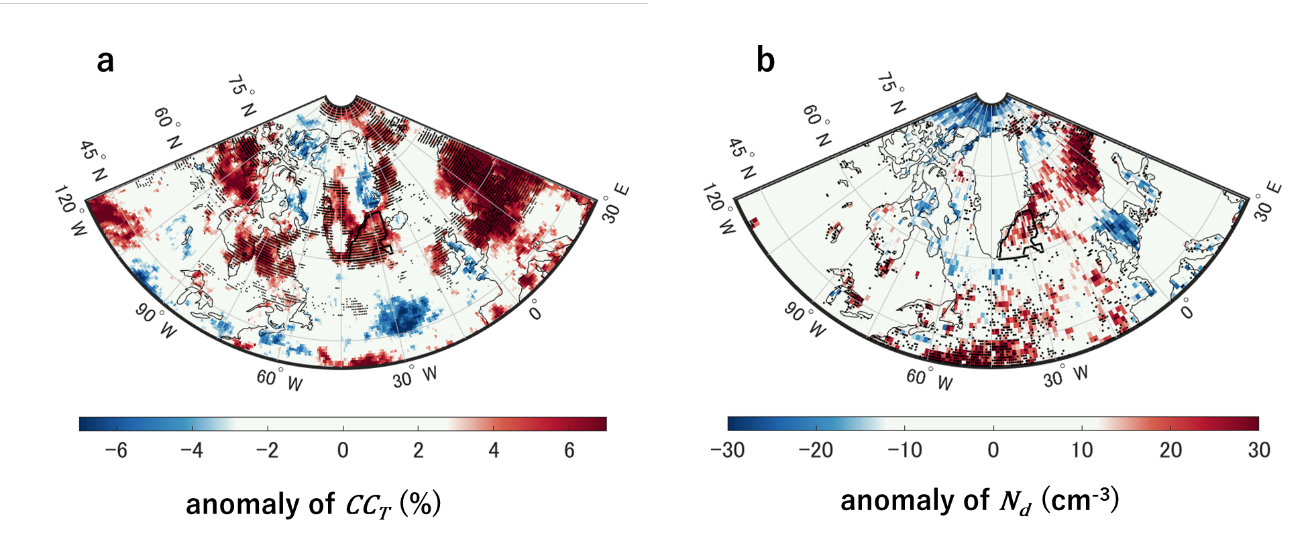


Figure S32. Geographical distribution of the anomaly of a, *CC_T_* and b, *N_d_* with a resolution of 0.5º in 1984 and 1987. The back dots mean denote that are statistically significantly confidence level correlate at 95% confidence level.


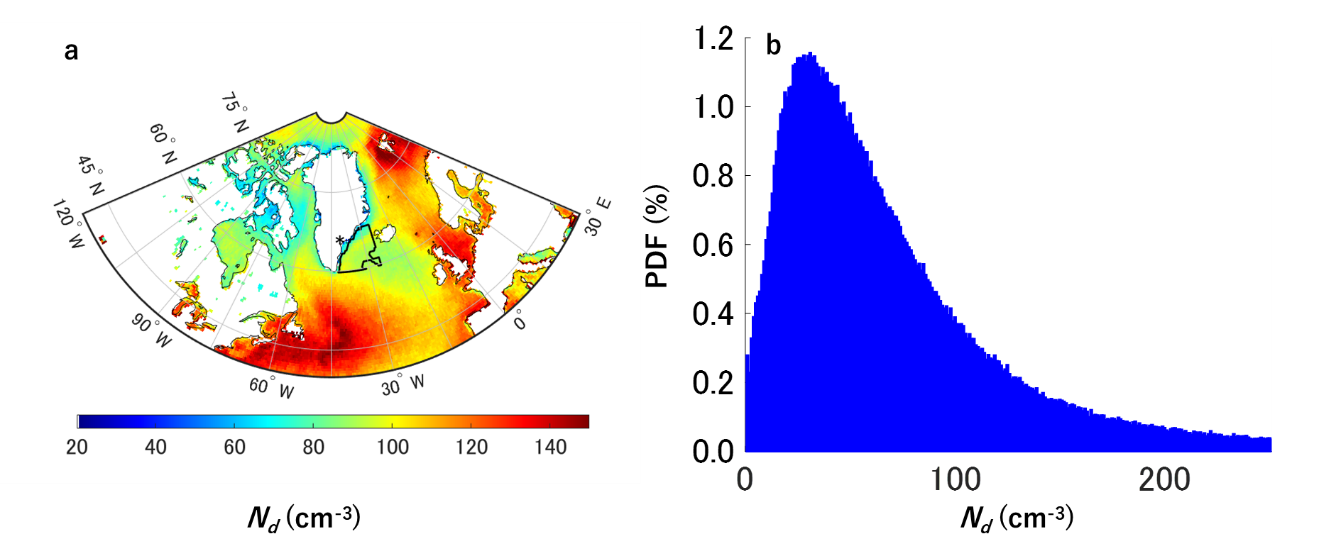


Figure S33 **a,** The geographical distribution of satellite-based $N_{d}$ with a resolution of 0.5º in summer from 1982 to 2014. Blackline and asterisk denote the target domain for the correlation analysis, and the SE-Dome site, respectively. **b,** The probability of calculated $N_{d}$in the target domain over the same period.

Table S1. Variables of cloud and meteorological properties used in this study. Open circles and crosses denote the data used and unused for investigation, respectively. Solid circle of $N_{d}$ was calculated using the Cloud_cci and ERA5 reanalysis data (see Methods). Uncertainties (relative values) related to cloud optical thickness and cloud particle size, those are necessary to the calculation of Nd, are noted in the text.
